# Conservative surgery vs hysterectomy for placenta accreta spectrum disorders: a systematic review and meta-analysis

**DOI:** 10.61622/rbgo/2026rbgo45

**Published:** 2026-08-20

**Authors:** Gabriel Moreira Lino, Pauliana Valéria Machado Galvão, Valda Lúcia Moreira Luna, George Alessandro Maranhão Conrado

**Affiliations:** 1 Universidade de Pernambuco Campus Serra Talhada Serra Talhada PE Brazil Universidade de Pernambuco, Campus Serra Talhada, Serra Talhada, PE, Brazil.; 2 Universidade de Pernambuco Faculdade de Ciências Médicas Recife PE Brazil Faculdade de Ciências Médicas, niversidade de Pernambuco, Recife, PE, Brazil.

**Keywords:** Placenta accreta, Hysterectomy, Fertility, Conservative treatment, Treatment outcome

## Abstract

**Objective:**

Resection and reconstruction of the uterus may be an alternative to hysterectomy in placenta accreta spectrum disorders. We aimed to review whether surgical and maternal outcomes were comparable between these techniques.

**Methods:**

Medline, Embase, and the Cochrane Library databases were searched until 1 January 2025. We included randomized controlled trials (RCT) and nonrandomized studies (NRSI) comparing conservative surgery, involving resection and reconstruction of the myometrium without placental removal, to hysterectomy in patients with placenta accreta, increta, or percreta. Two authors were responsible for independently screening the title and abstract, and the full texts of all identified records. We assessed the risk of bias with ROB-2 or ROBINS-I, and the quality of evidence with GRADE. The main analysis only included studies without a high risk of bias. A secondary non-inferiority Bayesian analysis was reported alongside the results.

**Results:**

We included one RCT and eleven nonrandomized studies (1586 women). Most women had FIGO PAS Grade 2 and 3a and with upper uterine segment involvement. Compared to hysterectomy, conservative surgery may result in a similar number of transfused packs of red blood cells (MD -0.70 packs, 95% CI -1.28 to -0.12; 1 RCT and 4 NRSI, 649 women; I2= 0%; low-certainty evidence), and a similar risk of bladder injury (RR 0.36, 95% CI 0.12 to 1.07; 1 RCT and 4 NRSI, 649 women; I2= 0%; low-certainty evidence). Excluding rare outcomes, the probability that conservative surgery is non-inferior to hysterectomy was greater than 90.0%.

**Conclusion:**

On the basis of low evidence, conservative surgery may not increase the risk of clinically important harm compared to hysterectomy, with the benefit of uterus preservation. Further RCTs are needed to reach definitive conclusions.

## Introduction

Until recently, hysterectomy was virtually the only management option for placenta accreta spectrum disorder (PAS). However, the prevalence of PAS has increased to affect approximately 1 in every 313 women undergoing cesarean section, prompting investigation into alternative strategies with reduced morbidity.^(1)^ Currently, leaving the placenta in situ is one of the most utilized alternatives to hysterectomy, although it requires high-resource settings and continuous, long-term, monitoring.^(2,3)^ In this context, uterus-preserving surgery (also referred to as one-step conservative surgery and triple P procedure, according to variations in the technique proposed by individual authors) has been developed to provide immediate surgical resolution of PAS during cesarean delivery.^(4)^

This technique involves removing the affected myometrium and reconstructing the uterine wall, with or without preventive surgical devascularization.^(2,4)^ However, it remains to be proven whether surgical outcomes are superior or similar to hysterectomy, specifically because of the challenging surgical anatomy and the inherent risk of hemorrhage and visceral organ injury. Therefore, this review aims to summarize the available evidence on this conservative surgical technique, comparing it to hysterectomy in patients diagnosed with PAS.

## Methods

### Protocol and registration

This systematic review was registered prospectively in the PROSPERO database, identifier CRD42024601117. We followed the PRISMA 2020 guidelines for the reporting of systematic reviews and meta-analyses.^(5)^

### Criteria for considering studies for this review

We considered randomized controlled trials (RCT) and nonrandomized studies of interventions comparing conservative surgery to hysterectomy in patients with placenta accreta, increta, or percreta. To be included, the surgical technique must have involved resection of abnormally implanted placenta and myometrium and reconstruction of the uterine wall. Therefore, we excluded studies that provided intraoperative resolution by manual removal of the placenta followed by vascular control procedures, such as uterine compressive sutures, tourniquets, and uterine devascularization. The following critical outcomes were considered: intraoperative blood loss, number of transfused packs of red blood cells or women needing transfusion, intraoperative visceral organ injury (bladder injury, bowel injury, ureter injury, and vascular injury), reoperation, long-term complications of surgery (fistula, premature ovarian insufficiency, urinary dysfunction, dysmenorrhea). The secondary outcomes of the review were: duration of surgery, duration of hospital stay, admission to the intensive care unit, and postoperative infection. Studies had to report at least one of the primary outcomes to be eligible for the review.

### Search methods for identification of studies

We searched Medline, Embase, and the Cochrane Library for published studies up to January 2025. The search strategy was adapted for each database (Supplementary Material 1). No restrictions were applied.

### Data collection and analysis

Two authors were responsible for independently screening the title and abstract of all identified records. When one of the authors considered the record eligible, both assessed the full text. Thereafter, they proceeded with selecting and extracting information about study characteristics, which were summarized on a cross-validated file.

### Risk of bias assessment in included studies

For the risk of bias assessment, two authors independently used the Cochrane risk-of-bias tool for randomized trials (RoB 2), version of August 2019.^(6)^ The ROBINS-I V2 tool, 2024, was used instead for nonrandomized studies of interventions.^(7)^ We evaluated the overall risk of bias in each study based on three primary outcomes (blood loss, transfusion, and intraoperative visceral organ injury). Dropouts, group conversions, or loss to follow-up below 5% were judged as low risk of bias.

### Synthesis methods

We used an inverse-variance random effects model, reporting dichotomous data as Relative Risk (RR) with 95% Confidence intervals (CI) and continuous outcomes as mean differences (MD) and 95% CI. Statistical heterogeneity was assessed by calculating tau (DerSimonian-Laird estimator and Jackson method for confidence interval of tau^2^), I^2^, and Cochran's Q; visual I^2^ variation that included clinically important differences indicated serious heterogeneity.^(8)^ We investigated publication bias with funnel plots and Egger's test. Studies that reported medians and interquartile ranges were transformed using an internal function of the meta package, following what was described by Luo and collaborators.^(9)^ We stratified our analyses based on the risk of bias. Primary results in every section only included studies with randomized design or that were not at high risk of bias. Subsequent analyses reported the estimated effect including the remaining studies, except when the results of both analyses were uncertain, making it unnecessary to report them separately. Analyses were conducted in R, with the packages meta and metafor.^(10,11)^

### Sensitivity analyses

To explore the certainty of the main results, we conducted a planned secondary noninferiority analysis using a Bayesian framework. Specifically, we assessed how integrating prior knowledge from observational studies not at serious or critical risk of bias would influence the interpretation of the randomized studies, which had limited statistical power due to modest sample sizes. We first synthesized the selected nonrandomized studies using a Bayesian meta-analysis with a non-informative prior (mean = 0, SD = 10,000), generating a posterior distribution that served as an informative prior for the subsequent analysis of the trial data. We then combined this prior with the likelihood derived from the randomized controlled trial to obtain a posterior estimate reflecting both sources of evidence. This reanalysis focused on critical clinical outcomes: number of packs of red blood cells transfused, intraoperative visceral injuries (including bladder and ureter), need for reoperation, and long-term surgical complications. The noninferiority margins were based on an absolute risk difference of 1% for the dichotomous outcomes. For the number of packs of red blood cells transfused, we determined 0.5 to 1 pack as the minimum number for clinical meaningfulness.

### Certainty of the evidence assessment

The overall certainty of the evidence for each outcome was assessed by two authors using the Grading of Recommendations, Assessment, Development and Evaluation (GRADE) handbook.^(12)^ The quality of the evidence was summarized in a Summary of Findings table generated in GRADEpro GDT. For the evaluation of imprecision, the update provided in 2022 was followed.^(13)^ The Review Information Size was determined using conventional power analysis (α = 0.05; β = 0.2), taking into account the range of standard deviations from randomized controlled trials and medium to low risk of bias studies.

## Results

### Results of the search

We initially identified 640 records in our search. Of these, 106 were deemed potentially eligible, and 12 (only one RCT) were ultimately included in the review. The primary reasons for exclusion were inadequate surgical techniques (manual placental removal or failure to report surgical methods), study design (such as case series, reports, reviews, editorials, and letters), absence of relevant outcome data specified in the review protocol, and not comparing conservative surgery to hysterectomy (such as comparing to manual removal of the placenta) (Figure 1).

**Figure 1:**
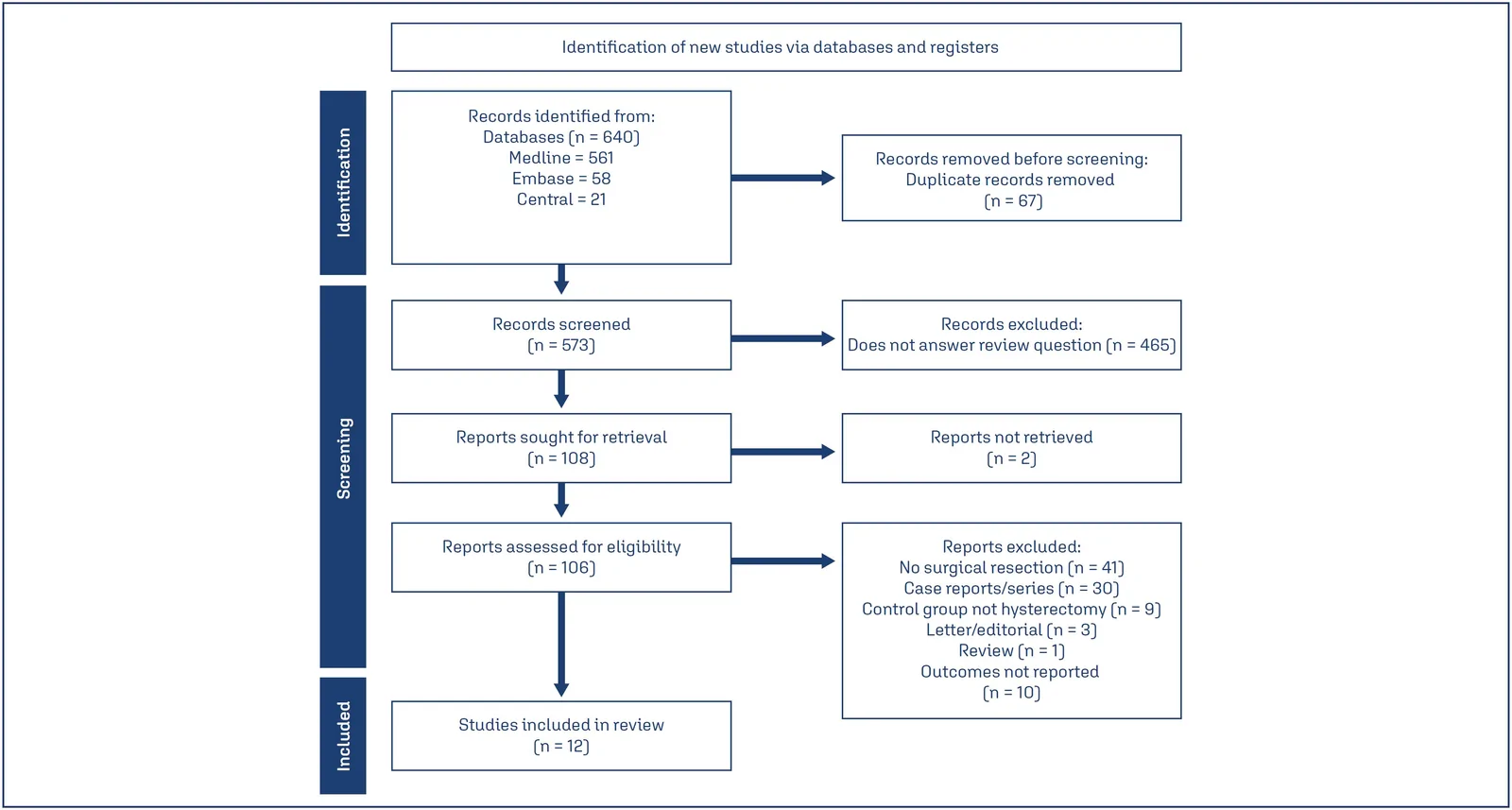
PRISMSA 2020 flow diagram of study selection

### Included studies

Chart 1 summarizes the characteristics of the included studies. One RCT and eleven non-randomized studies were included, four of which had prospective designs. Moreover, some studies reported the PAS FIGO clinical grading,^(14)^ with patients of every grade in both arms. The average maternal age ranged from 30 to 37 years, with most women having had one or two previous cesarean sections. Preventive vascular interventions before myometrial resection were utilized in three of the included studies. Further descriptions of methods, eligibility criteria, and participants are available in Table 1. In general, the most common eligibility criteria for uterine conservative surgery were: (1) at least 2 cm of healthy myometrium in the anterior uterine wall, (2) more than 50% of the uterine circumference without PAS involvement, and (3) the ability to safely dissect the bladder from the uterus. Most studies followed the surgical technique described as one-step conservative surgery.^(4)^

**Chart 1:** Summary included studies

Author (Year)	Country	Study design	Sample size (CS;HT)	PAS FIGO grading (n)		Maternal age (y)		Previous cesarean deliveries (n)		Preventive vascular intervention	Risk of bias
				CS	HT	CS	HT	CS	HT		
Nieto-Calvache et al. 2024^(15)^	Multileft	RCT, not blinded	60 (29;31)	Grade 1 2 Grade 2 18 Grade 3A 3 Grade 3B-C4	1 20 0 3	33.45±4.56	33.13 ±4.26	-	-	No	Moderate risk
Nieto-Calvache et al. 2023^(16)^	Multileft	NRSI, prospective design	75 (64;11)	-	-	33 (26−38)	31 (29−37)	-	-	No	Moderate risk
Paping et al. 2024^(17)^	Multileft	NRSI, retrospective design, prospectively-collected data	224 (38;186)	Grade 1 10 Grade 2 13 Grade 3A 14 Grade 3B-C 1	21 46 60 59	34 (30–37)	35 (31–39)	2 (1–3)	2 (1–2)	No	Moderate risk
Abouda et al. 2025^(18)^	Single left	NRSI, prospective design	12 (6;6)	-	-	37.17±2.93	33.67±3.07	4±0.89	2.83±0.98	No	Moderate risk
van Beekhuizen et al. 2021^(19)^	Multileft	NRSI, retrospective design, prospectively collected data	278 (26;252)	Grade 1 3 Grade 2 2 Grade 3A 18 Grade 3B-C 3	31 52 102 66	-	-	-	-	No	Moderate risk
Vuong et al. 2024^(20)^	Single left	NRSI, retrospective design	296 (217;79)			33.17 ± 4.77	33.68 ± 4.94	-	-	No	High risk
Aryananda et al. 2024^(21)^	Single left	NRSI, retrospective design	126 (91;35)	Grade 1 16 Grade 2 54 Grade 3A 15 Grade 3B-C 6	0 7 10 18	33 (22–46)	33 (22–42)	2 (1–3)	2 (1–3)	No	High risk
Aryananda et al. 2022^(22)^	Single left	NRSI, retrospective design	234 (200;34)	-	-	33 (19–44)	35 (26–41)			Yes: internal iliac artery ligation	High risk
Jafari et al. 2022^(23)^	Single left	NRSI, retrospective design	68 (44;24)	-	-	33.23 ± 4.72	32.54 ± 5.69	-	-	No	High risk
Durukan, et al. 2021^(24)^	Single left	NRSI, retrospective design	65 (23;42)	-	-	33.1 ± 5.4	34.1 ± 5.4	2.0 ± 1.1	2.1 ± 0.9	No	High risk
Üstünyurt 2020^(25)^	Single left	NRSI, retrospective design	97 (30;67)	-	-	31.1±5.4	33.2±4.7	-	-	Yes: Intrauterine balloon tamponade	High risk
Kilicci et al. 2018^(26)^	Single left	NRSI, retrospective design	51 (22;29)	-	-	32.3 ±5.3	32.45± 4.7	1.6 ±0.9	1.8 ±0.6	No	High risk

RCT: randomized controlled trial; NRSI: nonrandomized study of intervention

**Table 1:** Characteristics of included studies

Authors, ano	Methods	Participants	Intervention	Outcomes
Nieto-Calvache et al., 2024^(15)^	Multileft study, parallel-group design Randomization: Computer generated Blinding: No Drop-outs: Crossover of 9 participants in intervention group and 7 in hysterectomy group	Number of participants: 60 Power analysis for sample size: No Follow-up: 6 weeks postpartum Eligibility for conservative surgery: the bladder can be separated from the uterus, at least 2 cm of healthy myometrium caudal to the PAS area on the anterior uterine wall, and >50% of the uterine circumference free of PAS Exclusion criteria: aged < 18 years, active bleeding, no placenta previa, and no previous live children	Intervention: Conservative surgery (specific variation: One step surgery) Control group: hysterectomy Preventive vascular intervention: No	1. Intraoperative bleeding volume 2. Length of surgery 3. Length of hospitalization 4. RBCU transfusion 5. Number of RBCU transfused 6. Bladder injury 7. Surgical reintervention 8. Admission to intensive care unit 9. Adverse event
Nieto-Calvache et al., 2023^(16)^	Multileft study, parallel-group, prospective non-randomized design Randomization: No Blinding: No Drop-outs: Not reported	Number of participants: 75 Power analysis for sample size: No Eligibility for conservative surgery: the bladder can be separated from the uterus, at least 2 cm of healthy myometrium caudal to the PAS area on the anterior uterine wall, and >50% of the uterine circumference free of PAS Exclusion criteria: Patients with severe vesicouterine fibrosis (type 4 PAS on the topographic classification) or lower lateral placental involvement (low parametrial invasion or type 2 lower topographic classification) were excluded as, in those cases, it is not possible to perform OSCS	Intervention: Conservative surgery (specific variation: One step surgery) Control group: hysterectomy Preventive vascular intervention: No	1. Intraoperative bleeding volume 2. Length of surgery 3. Length of hospitalization 4. RBCU transfusion 5. Number of RBCU transfused 6. Bladder injury 7. Surgical reintervention
Paping et al. 2024^(17)^	Multileft, parallel-group, prospectively collected-data, retrospective analysis, non-randomized design Randomization: Computer generated Blinding: No Drop-outs: In the intention-to-treat analysis, 26 (46.4%) of prospectively assigned conservative surgery cases were instead managed by hysterectomy. Conversely, only 5 cases (3%) of hysterectomy were managed by conservative management.	Number of participants: 224 Power analysis for sample size: No Eligibility for conservative surgery: Based on each left's protocol. Exclusion criteria: -	Intervention: Conservative surgery Control group: hysterectomy (third arm was leaving the placenta-in-situ, not included in the review) Preventive vascular intervention: No	1. Intraoperative bleeding volume 2. Length of surgery 3. Length of hospitalization 4. RBCU transfusion 5. Number of RBCU transfused 6. Bladder injury 7. Surgical reintervention 8. Admission to intensive care unit 9. Adverse event
Abouda et al., 2025^(18)^	Single-left study, parallel-group, prospective, non-randomized design Randomization: No Blinding: No Drop-outs: 0	Number of participants: 12 Power analysis for sample size: No Eligibility for conservative surgery: at least 2 cm of healthy myometrium caudal to the PAS area on the anterior uterine wall, and >50% of the uterine circumference free of PAS Exclusion criteria: successful manual removal of the placenta without significant bleeding and the absence of histologic confirmation of PAS postoperatively	Intervention: Conservative surgery (specific variation: One-step surgery) Control group: hysterectomy Preventive vascular intervention: No	1. Intraoperative bleeding volume (Hb change) 2. Number of pRBC transfused 3. Duration of surgery
van Beekhuizen et al., 2021^(19)^	Multileft study, parallel-group, prospectively-collected data, retrospective analysis, non-randomized design Randomization: No Blinding: No Drop-outs: Not informed	Number of participants: 278 Power analysis for sample size: No Eligibility for conservative surgery: Not informed Exclusion criteria: Not informed	Intervention: Conservative surgery Control group: hysterectomy (leaving the placenta in situ was the third arm, and extirpative management was the fourth arm, both not included in the review) Preventive vascular intervention: No	1. Intraoperative bleeding volume 2. Number of RBCU transfused 3. ICU admission 4. Intraoperative visceral organ injury 5. Reoperation
Vuong et al., 2024^(20)^	Single-left study, parallel-group, retrospective, non-randomized design Randomization: No Blinding: No Drop-outs: Retrospective design Observations: 13 hysterectomies and 25 conservative surgeries were considered emergency surgeries	Number of participants: 296 Power analysis for sample size: No Eligibility for conservative surgery: pregnant women with a diagnosis of PAS at more than 28 weeks of pregnancy and more than 2 cm of healthy myometrium Exclusion criteria: emergency management such as massive bleeding immediately before surgery, and uterine rupture. If a hysterectomy was indicated after the steps of the MOSCUS procedure proved insufficient, the patient was also excluded from the study. Other exclusion criteria included missing necessary data on the patient's file and the patient not receiving pathology results.	Intervention: Resection surgery + preventive bilateral uterine artery ligation + B-lynch suture Control group: hysterectomy Preventive vascular intervention: No	1. Intraoperative bleeding volume 2. Length of surgery 3. Length of hospitalization 4. RBCU transfusion 5. Number of RBCU transfused 6. Bladder injury 7. Surgical reintervention 8. Admission to intensive care unit
Aryananda et al., 2024 ^(21)^	Single-left study, parallel-group, retrospective, non-randomized design Randomization: No Blinding: No Drop-outs: Retrospective design Observations: 6 hysterectomies and 14 conservative surgeries were considered emergency surgeries	Number of participants: 126 Power analysis for sample size: No Eligibility for conservative surgery: PAS topography above the bladder trigone (supero-anterior or upper parametrium PAS) was deemed suitable for OSCS. All women with a previous cesarean section and a placenta previa in the index pregnancy were identified as having risk factors for PAS and were screened by ultrasound. Exclusion criteria: dehiscence of the uterine scar without abnormally adherent placenta, no signs of increased vascularity on the uteroplacental surface and uterine serosa during the surgery, and women in an unstable hemodynamic condition before surgery without adequate pre-surgical ultrasound. Emergency PAS cases with vaginal bleeding or uterine rupture but in a stable hemodynamic condition were not excluded if ultrasound examinations could be performed.	Intervention: Conservative surgery (variation: one-step surgery) Control group: hysterectomy Preventive vascular intervention: No	1. Intraoperative bleeding volume 2. RBCU transfusion 3. Number of RBCU transfused
Aryananda et al., 2022^(22)^	Single-left study, parallel-group, retrospective, non-randomized design Randomization: No Blinding: No Drop-outs: Retrospective design	Number of participants: 234 Power analysis for sample size: No Eligibility for conservative surgery: PAS topography above the bladder trigone (supero-anterior or upper parametrium PAS) was deemed suitable for OSCS. All women with a previous cesarean section and a placenta previa in the index pregnancy were identified as having risk factors for PAS and were screened by ultrasound. Exclusion criteria: dehiscence of the uterine scar without abnormally adherent placenta, no signs of increased vascularity on the uteroplacental surface and uterine serosa during the surgery, and women in an unstable hemodynamic condition before surgery without adequate pre-surgical ultrasound. Emergency PAS cases with vaginal bleeding or uterine rupture but in a stable hemodynamic condition were not excluded if ultrasound examinations could be performed.	Intervention: Conservative surgery (variation: MOSCUS) Control group: hysterectomy Subgroup: Resection surgery + bilateral iliac artery ligation Preventive vascular intervention: No. Only a subgroup of patients underwent bilateral internal iliac artery ligation	1. Intraoperative bleeding volume 2. Length of surgery 3. Length of hospitalization 4. RBCU transfusion 5. Number of RBCU transfused 6. Bladder injury 7. Surgical reintervention 8. Admission to intensive care unit
Jafari et al., 2022^(23)^	Single-left study, parallel-group, retrospective, non-randomized design Randomization: No Blinding: No Drop-outs: Retrospective design	Number of participants: 68 Power analysis for sample size: No Eligibility for conservative surgery: Not informed Exclusion criteria: Not informed	Intervention: Conservative surgery Control group: hysterectomy Preventive vascular intervention: No	1. Hospital stay 2. RBCU transfusion 3. Duration of surgery
Durukan et al., 2021^(24)^	Single-left study, parallel-group, retrospective, non-randomized design Randomization: No Blinding: No Drop-outs: Retrospective design	Number of participants: 65 Power analysis for sample size: No Eligibility for conservative surgery: Not informed Exclusion criteria: Not informed	Intervention: Conservative surgery Control group: hysterectomy Preventive vascular intervention: No	1. Intraoperative bleeding volume 2. RBCU transfusion 3. ICU stay
Üstünyurt, 2020^(25)^	Single-left study, parallel-group, retrospective, non-randomized design Randomization: No Blinding: No Drop-outs: Retrospective design Observations: Statistical analysis was adjusted for confounders such as age, gravidity, parity, previous cesarean delivery, curettage, and myomectomy	Number of participants: 97 Power analysis for sample size: No Eligibility for conservative surgery: sonographic and intraoperative findings are appropriate in patients who prefer the preservation of the uterus, uterine sparing surgery is performed Exclusion criteria: Conservative surgery is not performed in cases with invasion into the parametrium and/ or cervix and invasion of more than 50% of the anterior surface of the uterus	Intervention: Conservative surgery Control group: hysterectomy Preventive vascular intervention: No	1. Intraoperative bleeding volume 2. RBCU transfusion 3. Number of RBCU transfused 4. Duration of surgery 5. Duration of hospital stay 6. ICU admission 7. Intraoperative visceral organ injury
Kilicci et al., 2018^(26)^	Single-left study, parallel-group, retrospective, non-randomized design Randomization: No Blinding: No Drop-outs: Retrospective design	Number of participants: 51 Power analysis for sample size: No Eligibility for conservative surgery: (a) Presence of an uninvolved area close to uterine arteries, (b) Applicability of urinary bladder dissection from anterior uterine wall Exclusion criteria: Patients undergoing urgent Cesarean section	Intervention: Conservative surgery Control group: hysterectomy Preventive vascular intervention: No	Number of RBCU transfused Duration of surgery Duration of hospital stay

### Risk of bias of included studies

An overview of the risk of bias is provided in Chart 1, and the detailed description is presented in Table 2. The included RCT was classified as having a moderate risk of bias due to the lack of blinding and important patient crossover/drop-out. Nearly all non-randomized studies were considered at high risk of bias, as patient selection was often at serious risk of bias. Additionally, few studies reported potential confounding variables or adjusted their analyses as necessary. An investigation into publication bias revealed that retrospective studies, which were at high risk of bias, generally reported higher mean differences with larger standard errors compared to studies with moderate to low risk of bias (Figure S1). However, Egger's Test did not detect significant publication bias affecting the analysis (p-value = 0.20).

**Table 2:** Bias assessment

Authors, ano	Risk of bias	Bias	Author's judgement	Supporting information
Nieto-Calvache et al., 2024^(15)^	Rob 2	Random sequence generation (selection bias)	Low risk	Computer generated
		Allocation concealment (selection bias)	Low risk	An appropriate analysis was used to estimate the effect of assignment to intervention
		Incomplete outcome data (attrition bias)	Low risk	Outcome data were available for all, or nearly all, randomized participants
		Measurement of the outcome	Some concerns	Open-label study. It is unlikely that assessment of the surgical outcomes was influenced by knowledge of intervention received.
		Selective reporting (re-porting bias)	Low risk	Study conducted according to a prospectively registered protocol
Nieto-Calvache et al., 2023^(16)^		Bias due to confounding	Some concerns	Comparable baseline characteristics, BMI and number of previous cesareans not reported. Adjusted analysis not performed.
		Bias in the selection of participants into the study	Some concerns	Prospectively recruited study, however, intervention could change based on intraoperative assessment.
		Bias in classification of the intervention	Low risk	Well defined intervention status.
		Bias due to deviations from intended interventions	Low risk	There weren't substantial deviations.
		Bias due to missing outcome data	Some concerns	Complete case analysis.
		Bias in measurement of the outcome	Some concerns	The methods of outcome assessment were comparable across intervention groups and outcome measure is only minimally affected by knowledge of the intervention
		Bias in selection of the reported result	Some concerns	There was no protocol. However, there appears to be no issues with multiple analyses or subgroups and nearly all relevant surgical outcomes were reported
Paping et al., 2024^(17)^	ROBINS-I	Bias due to confounding	Some concerns	Comparable baseline characteristics, BMI not reported. Adjusted analysis not performed.
		Bias in the selection of participants into the study	Low risk	Prospectively recruited study. "Participants were first grouped based on the actual treatment performed after intraoperative assessment and staging. A second analysis was performed using an intention-to-treat analysis, whereby participants were grouped based on the preoperative planned surgery".
		Bias in classification of the intervention	Low risk	Well defined intervention status.
		Bias due to deviations from intended interventions	Low risk	There weren't substantial deviations.
		Bias due to missing outcome data	Low risk	Nearly all patients had outcome data. Excluded cases were not related to lack of data.
		Bias in measurement of the outcome	Some concerns	The methods of outcome assessment were comparable across intervention groups and outcome measure is only minimally affected by knowledge of the intervention
		Bias in selection of the reported result	Some concerns	There was no protocol. However, there appears to be no issues with multiple analyses or subgroups and nearly all relevant surgical outcomes were reported
Abouda et al., 2024^(18)^	ROBINS-I	Bias due to confounding	Some concerns	Comparable baseline characteristics. Adjusted analysis not performed.
		Bias in the selection of participants into the study	Low risk	Prospectively recruited study according to a prespecified protocol.
		Bias in classification of the intervention	Low risk	Well defined intervention status.
		Bias due to deviations from intended interventions	Low risk	There weren't substantial deviations.
		Bias due to missing outcome data	Low risk	Outcome data was complete.
		Bias in measurement of the outcome	Low risk	The methods of outcome assessment were comparable across intervention groups and outcome measure is only minimally affected by knowledge of the intervention. A prespecified protocol defined how outcome would be measured before the start of the study.
		Bias in selection of the reported result	Some concerns	Study conducted according to protocol.
van Beekhuizen et al., 2021^(19)^	ROBINS-I	Bias due to confounding	Some concerns	Comparable baseline characteristics, BMI and maternal age not reported. Adjusted analysis not performed.
		Bias in the selection of participants into the study	Some concerns	Prospectively recruited study, however, intervention could change based on intraoperative assessment.
		Bias in classification of the intervention	Low risk	Well defined intervention status.
		Bias due to deviations from intended interventions	Low risk	There weren't substantial deviations.
		Bias due to missing outcome data	Low risk	Nearly all patients had outcome data. Excluded cases were not related to lack of data.
		Bias in measurement of the outcome	Some concerns	The methods of outcome assessment were comparable across intervention groups and outcome measure is only minimally affected by knowledge of the intervention
		Bias in selection of the reported result	Some concerns	There was no protocol. However, there appears to be no issues with multiple analyses or subgroups and nearly all relevant surgical outcomes were reported
Vuong et al., 2024^(20)^	ROBINS-I	Bias due to confounding	High	Retrospective analysis, not adjusted.
		Bias in the selection of participants into the study	High	Retrospective recruited study, intervention could change based on intraoperative assessment.
		Bias in classification of the intervention	Low risk	Well defined intervention status.
		Bias due to deviations from intended interventions	Low risk	There weren't substantial deviations.
		Bias due to missing outcome data	Some concerns	Complete case analysis.
		Bias in measurement of the outcome	Some concerns	The methods of outcome assessment were comparable across intervention groups and outcome measure is only minimally affected by knowledge of the intervention
		Bias in selection of the reported result	Some concerns	There was no protocol. However, there appears to be no issues with multiple analyses or subgroups and nearly all relevant surgical outcomes were reported
Aryananda et al., 2023^(21)^	ROBINS-I	Bias due to confounding	High	Retrospective analysis, not adjusted.
		Bias in the selection of participants into the study	High	Retrospective recruited study, intervention could change based on intraoperative assessment.
		Bias in classification of the intervention	Some concerns	A substantial number of failed conservative management may have crossed over to hysterectomy, compromising the analysis
		Bias due to deviations from intended interventions	Low	There weren't substantial deviations.
		Bias due to missing outcome data	Some concerns	Complete case analysis.
		Bias in measurement of the outcome	Some concerns	The methods of outcome assessment were comparable across intervention groups and outcome measure is only minimally affected by knowledge of the intervention.
		Bias in selection of the reported result	High	There was no protocol. Important outcomes were not reported.
Aryananda et al., 2022^(22)^	ROBINS-I	Bias due to confounding	High	Retrospective analysis, not adjusted.
		Bias in the selection of participants into the study	Some concerns	Retrospective recruited study, but consecutive cases were included. A flow diagram of patient selection is provided. Selection criteria was reasonable to assume high risk was not present (only cases of upper trigonal bladder invasion were included in hysterectomy and conservative surgery).
		Bias in classification of the intervention	Low risk	Well defined intervention status.
		Bias due to deviations from intended interventions	Low risk	There weren't substantial deviations.
		Bias due to missing outcome data	Some concerns	Complete case analysis.
		Bias in measurement of the outcome	Some concerns	The methods of outcome assessment were comparable across intervention groups and outcome measure is only minimally affected by knowledge of the intervention
		Bias in selection of the reported result	Some concerns	There was no protocol. However, there appears to be no issues with multiple analyses or subgroups and nearly all relevant surgical outcomes were reported
Jafari et al., 2022^(23)^	ROBINS-I	Bias due to confounding	High	Retrospective analysis, not adjusted.
		Bias in the selection of participants into the study	High	Retrospective recruited study, intervention could change based on intraoperative assessment.
		Bias in classification of the intervention	Low	Classification was well defined.
		Bias due to deviations from intended interventions	Low	There weren't substantial deviations.
		Bias due to missing outcome data	Some concerns	Complete case analysis.
		Bias in measurement of the outcome	Some concerns	The methods of outcome assessment were comparable across intervention groups and outcome measure is only minimally affected by knowledge of the intervention.
		Bias in selection of the reported result	Some concerns	There was no protocol. Nearly all important outcomes were reported.
Durukan et al., 2021^(24)^	ROBINS-I	Bias due to confounding	High	Retrospective analysis, not adjusted.
		Bias in the selection of participants into the study	High	Retrospective recruited study, intervention could change based on intraoperative assessment.
		Bias in classification of the intervention	Some concerns	Conservative surgery may have been attempted in hysterectomy cases.
		Bias due to deviations from intended interventions	Low	There weren't substantial deviations.
		Bias due to missing outcome data	High	No information is provided
		Bias in measurement of the outcome	Some concerns	The methods of outcome assessment were comparable across intervention groups and outcome measure is only minimally affected by knowledge of the intervention.
		Bias in selection of the reported result	High	There was no protocol. Important outcomes were not reported.
Üstünyurt, 2020^(25)^	ROBINS-I	Bias due to confounding	Some concerns	Retrospective analysis, adjusted.
		Bias in the selection of participants into the study	High	Retrospective recruited study, intervention could change based on intraoperative assessment.
		Bias in classification of the intervention	Low	Classification was well defined.
		Bias due to deviations from intended interventions	Low	There weren't substantial deviations.
		Bias due to missing outcome data	Low	Patients were not excluded based on missing outcome data
		Bias in measurement of the outcome	Some concerns	The methods of outcome assessment were comparable across intervention groups and outcome measure is only minimally affected by knowledge of the intervention.
		Bias in selection of the reported result	Low	Nearly all outcomes of interest reported. there appears to be no selection based on outcome, intervention, multiple analyses, or different subgroups.
Kilicci et al., 2018^(26)^	ROBINS-I	Bias due to confounding	High	Retrospective analysis, not adjusted.
		Bias in the selection of participants into the study	Some concerns	Retrospective recruited study, intervention could change based on intraoperative assessment. Selection criteria was adequate.
		Bias in classification of the intervention	Low	Classification was well defined.
		Bias due to deviations from intended interventions	Low	There weren't substantial deviations.
		Bias due to missing outcome data	Some concerns	Complete case analysis.
		Bias in measurement of the outcome	Some concerns	The methods of outcome assessment were comparable across intervention groups and outcome measure is only minimally affected by knowledge of the intervention.
		Bias in selection of the reported result	Some concerns	Not all outcomes of interest reported.

### Synthesis of results

#### Intraoperative blood loss

Conservative surgery may result in similar intraoperative blood loss compared to hysterectomy (MD -320.89 mL, 95% CI -574.95 to -66.82; 1 RCT and 3 NRSI, 637 women; I^2^= 14%; low-certainty evidence). By including studies at high risk of bias, the difference in blood loss is larger, but of lower certainty (MD -497.33 mL, 95% CI -755.37 to -226.17; 1 RCT and 7 NRSI; I^2^= 73%; very low-certainty evidence) (Figure 2).

**Figure 2:**
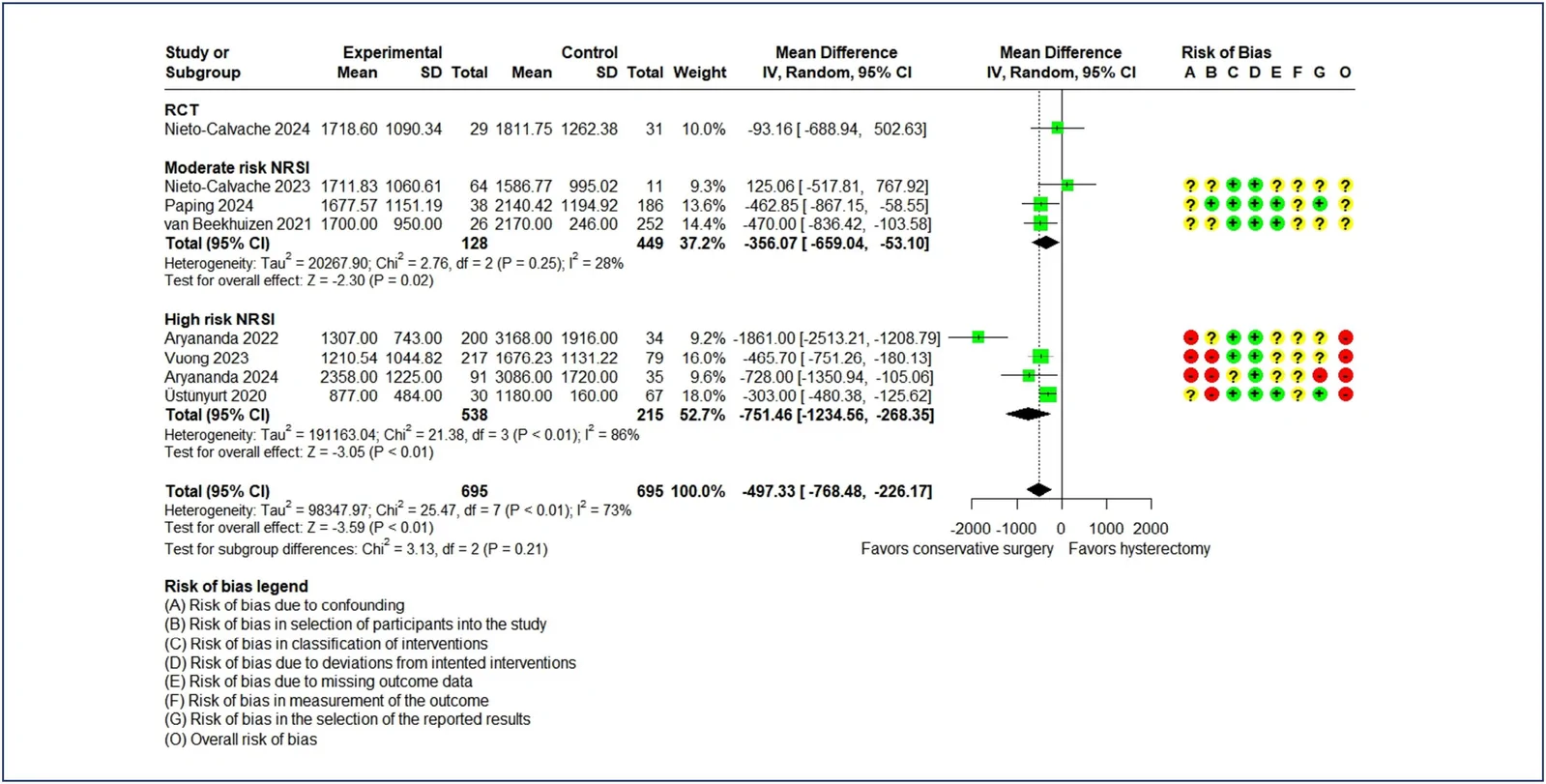
Comparison of uterine conservative surgery vs hysterectomy on intraoperative blood loss in women with placenta accreta spectrum disorders

#### Number of transfused packs of red blood cells (pRBCs) and number of women needing transfusion

The number of transfused pRBCs per patient may be similar in conservative surgery compared to hysterectomy (MD -0.70 packs, 95% CI -1.28 to -0.12; 1 RCT and 4 NRSI, 649 women; I^2^= 0%; low-certainty evidence). This benefit could be slightly larger (MD -1.15 packs, 95% CI -1.64 to -0.65; 1 RCT and 9 NRSI; I^2^= 20%; very low-certainty evidence) (Figure 3). Further, assuming a non-inferiority margin of 0.5 pRBCs, the probability that conservative surgery is non-inferior to hysterectomy is 95.4% (Figures S2 and S3).

**Figure 3:**
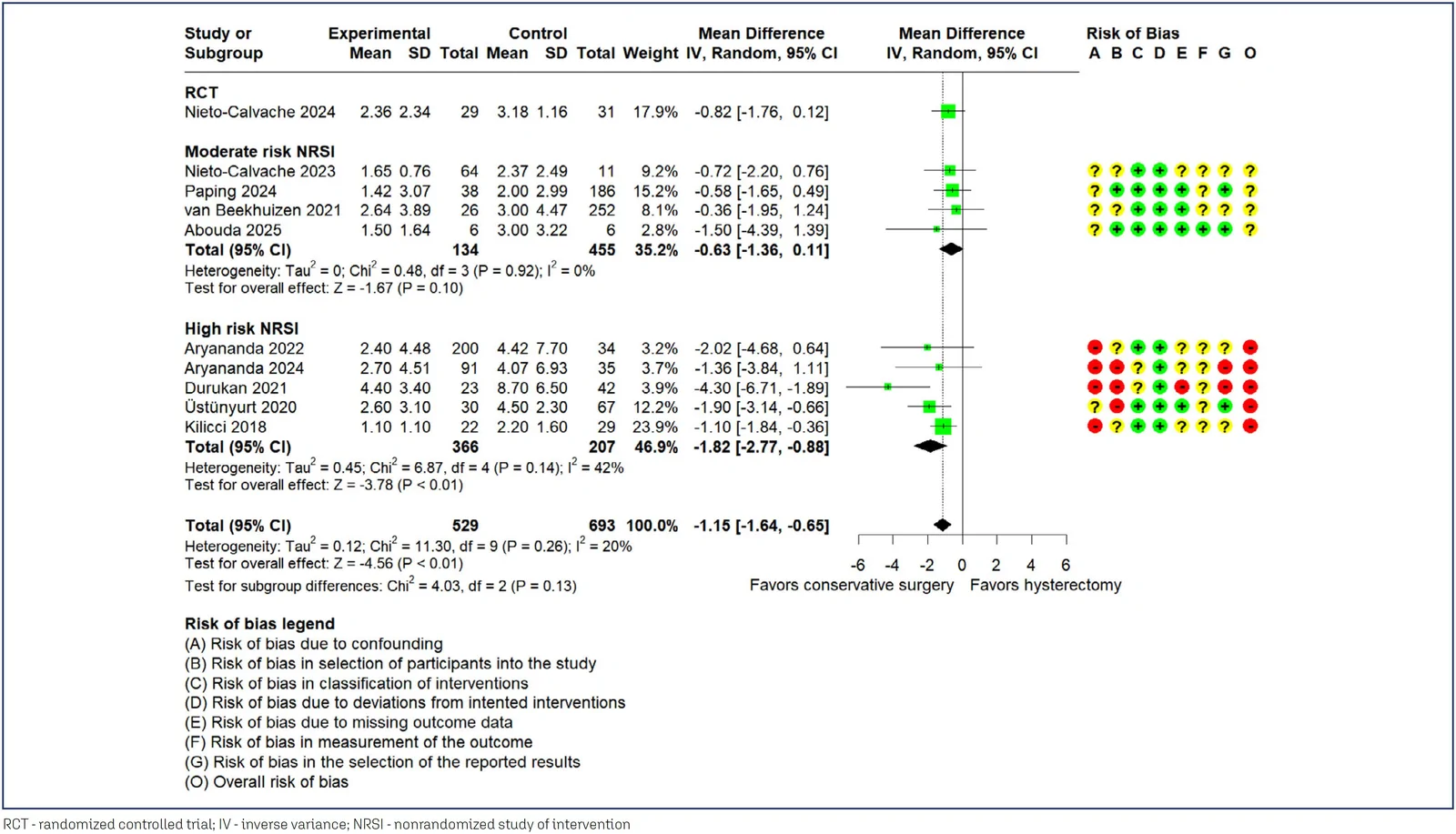
Comparison of uterine conservative surgery to hysterectomy on the number of transfused packs of red blood cells per patient in women with placenta accreta spectrum disorders.

Moreover, there may be a similar number of patients needing blood transfusion (RR 0.87, 95% CI 0.67 to 1.13; 1 RCT and 1 NRSI, 135 women; I^2^= 0%; low-certainty evidence) or fewer women will require transfusion (RR 0.82, 95% CI 0.74 to 0.91; 1 RCT and 4 NRSI; I^2^= 0%; very low-certainty evidence).

#### Intraoperative visceral organ injury

Few cases of bladder injury were reported, therefore, we found similar event rates for both groups (RR 0.36, 95% CI 0.12 to 1.07; 1 RCT and 4 NRSI, 649 women; I^2^= 0%; low-certainty evidence). Potentially, the risk of bladder injury could be lower with conservative surgery (RR 0.32, 95% CI 0.19 to 0.54; 1 RCT and 6 NRSI; I^2^= 0%; very low-certainty evidence) (Figure 4). Assuming a risk difference of 1% as the threshold for noninferiority, the probability was 71.5% (Figures S4 and S5).

**Figure 4:**
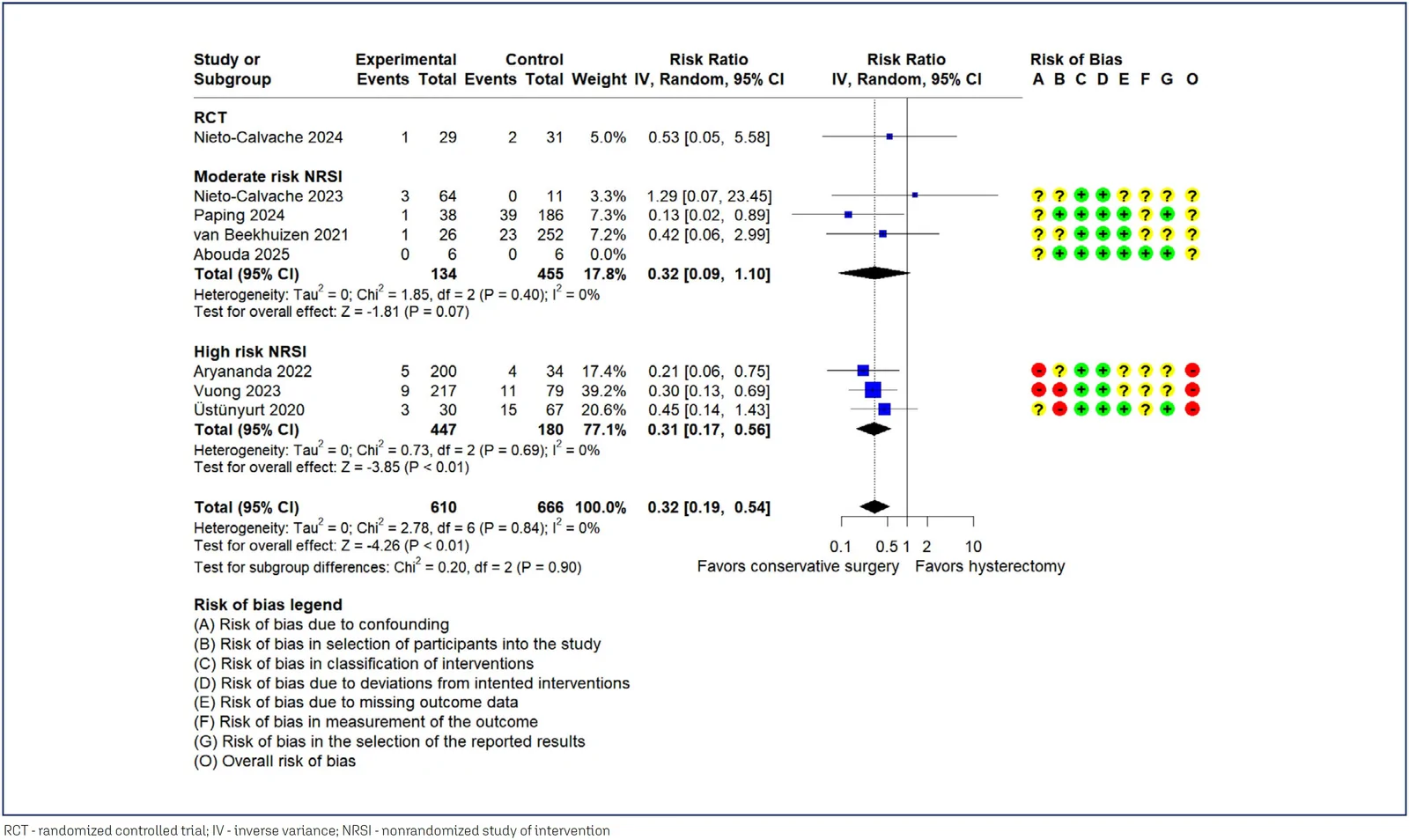
Comparison of uterine conservative surgery to hysterectomy on bladder injury in women with placenta accreta spectrum disorders

In the two studies that investigated ureter injuries, no cases were observed in either arm (2 NRSI, 87 women; very low-certainty evidence). Likewise, no cases of bowel injury were reported with conservative surgery, and seven were reported with hysterectomy (RR 0.17, 95% CI 0.02 to 1.65; 3 NRSI; I^2^= 17%; very low-certainty evidence).

#### Reoperation

Very few cases of reoperation were reported in both groups, hence, the pooled estimate was very wide (RR 0.50, 95% CI 0.07 to 3.51; 1 RCT and 3 NRSI; I^2^= 0%; very low-certainty evidence)(Figure S6). The non-inferiority analysis was uninformative due to low event rates (Figures S7 and S8).

#### Long-term complications of surgery

Vesicovaginal fistula formation was the only long-term complication reported across studies. As previously observed, this outcome was rare in both groups, leading to a wide effect estimate (RR 1.40, 95% CI 0.16 to 12.08; 1 RCT and 1 NRSI, 294 women; I^2^ = 0%; very low-certainty evidence)(Figure S9). The non-inferiority analysis was uninformative due to low event rates (Figures S10 and S11).

#### Duration of surgery

There was little to no difference in the duration of surgery (MD -36.31 minutes, 95% CI -73.06 to 0.43; 1 RCT and 2 NRSI, 147 women; I^2^= 54%; low-certainty evidence). Very uncertainly, conservative surgery could be shorter, however, high heterogeneity limits the confidence in this possibility (MD -26.45 minutes, 95% CI -45.35 to -7.56; 1 RCT and 6 NRSI; I^2^= 89%; very low-certainty evidence) (Figure 5).

**Figure 5:**
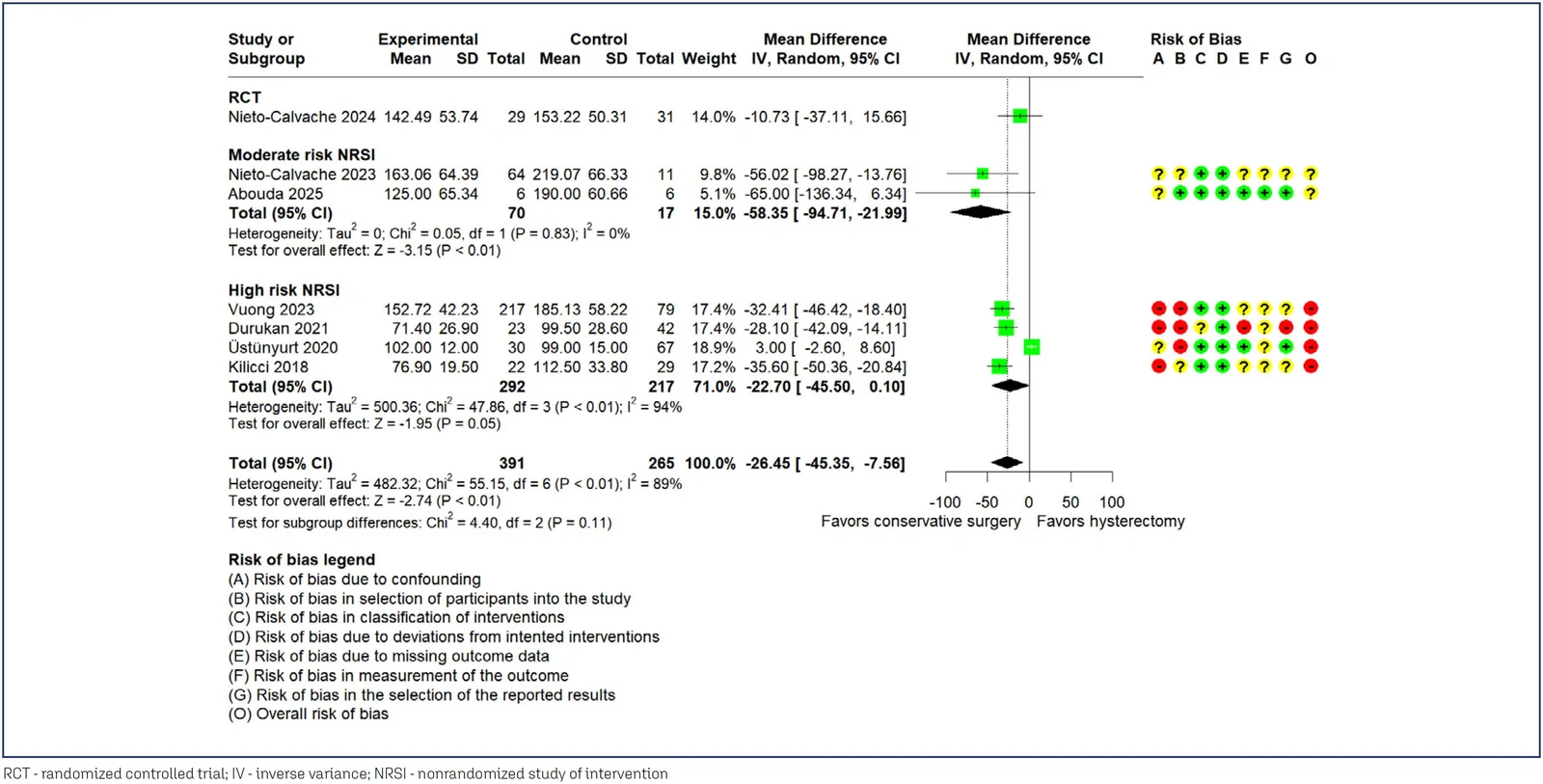
Comparison of uterine conservative surgery to hysterectomy on the duration of surgery in women with placenta accreta spectrum disorders

#### Duration of hospital stay

There was little to no difference in the duration of hospital stay (MD -0.39 days, 95% CI -0.87 to 0.08; 1 RCT and 4 NRSI; I^2^=33%; very low-certainty evidence). This could depend on institutional protocols and may not relate to the surgeries investigated.

#### Admission to the intensive care unit (ICU)

There was little to no difference in the risk of ICU admission (RR 1.11, 95% CI 0.83 to 1.47; 1 RCT and 4 NRSI; I^2^= 0%; very low-certainty evidence).

#### Certainty of evidence

The certainty of the evidence ranged from low to very low, according to the GRADE assessment (Table 2). Whenever the analysis included nonrandomized studies, the evidence was limited to low. To overcome these limitations on evidence, the Review Information Size (RIS) necessary to not downgrade for imprecision, for equally divided randomized controlled trials, would be: 166 to 594 participants for pRBC, and 210 to 4908 participants for blood loss, assuming moderate to small effects for the outcomes. Conversely, more rare outcomes (visceral organ injury and ICU admission) would need far greater sample sizes, which might not be feasible.

## Discussion

### Summary of main results

Conservative surgery has the advantage of preserving the uterus with surgical outcomes that were similar to hysterectomy in our analyses, in light of low-certainty evidence. Visceral organ injury, reoperation, and long-term complications were fairly rare, while blood loss was similar. In this regard, there is a high probability that conservative surgery is non-inferior to hysterectomy. However, PAS topography was an influential factor in the success of conservative surgery, with studies mostly including Grade 2 and 3a pathologies, and reporting more favourable outcomes with upper segment rather than lower segment involvement.

### Limitations of the evidence included in the review

The most important limitation of our study is the inclusion of nonrandomized studies of interventions. Major limitations that future trials on PAS would face are the low incidence of PAS, which requires longer recruitment and results in smaller samples, the long-term follow-up needed to determine patient-centered outcomes, and the specific placenta topography that limits the number of eligible women to be recruited. Fundamentally, nonrandomized studies are better at evaluating potential harm than potential benefit.^(27)^ Inherent consequences of results drawn from these studies are uncontrolled confounding and suboptimal outcome reporting. Even after excluding high-risk studies from the main analysis, the confidence in the estimates provided was low. If only the data of the randomized trial were analyzed, the small sample size and wide confidence intervals would make it unreasonable to assume any given effect for conservative surgery. As such, we provided a noninferiority analysis using the nonrandomized studies as a moderately informative prior to the randomized evidence, in an attempt to minimize potential biases by making the randomized data more influential. Nevertheless, the results should be interpreted in the context of low certainty evidence. Additionally, not all patients can be considered for conservative surgery, as there are topographical constraints to this technique, making patient selection crucial for the success of this surgical approach. Another limitation was the lack of patient-driven outcomes in the included studies. However, it could be argued that uterus preservation (with reasonable safety) would be the most prominent outcome for patients.

### Agreements and disagreements with other studies or reviews

Subsequent pregnancies after any conservative management of PAS (placenta left in situ and surgical resection) are associated with higher maternal morbidity, with one in every four pregnancies having adverse maternal outcomes.^(28)^ Whether nonoperative and operative management lead to different outcomes is unclear. In one retrospective study including both conservative surgery and manual removal of the placenta with compression sutures, out of 75 pregnancies, eight resulted in cesarean scar or ectopic pregnancies.^(29)^ Comparatively, another retrospective study, which only included pregnancies following conservative surgery, did not report any case of PAS in 202 pregnancies, and only one hysterectomy due to patient preference.^(30)^ Such findings should be interpreted in the context of very low-certainty evidence; however, they suggest that operative management may be superior in this regard. Our results add substantially to the initial assessment from FIGO in 2018, in which it was suggested that leaving the placenta in situ was a valid management option for women desiring to preserve fertility while conservative surgery remained unclear^2^. Here, we present both randomized and prospective nonrandomized data suggesting the safety of conservative surgery, especially in upper uterine segment topographies. In theory, considering both nonsurgical, surgical-resection, and hysterectomy for PAS would be ideal, as these approaches have different shortcomings and eligibility criteria. Individualized management of PAS should consider equally expertise with available techniques, infrastructure, technology, patient preferences, and social context.^(31)^

Moreover, there are many variations of conservative surgery, which include prophylactic balloon occlusion or major vascular intervention (such as the triple P procedure).^(32)^ However, many of these studies did not compare their interventions to hysterectomy and, therefore, were excluded from our review. The studies that were eventually included were not randomized, and almost all had a high risk of bias, making it impossible to derive any conclusions about prophylactic vascular interventions. In previous investigations, meta-analyses and randomized controlled trials reported conflicting results for prophylactic balloon occlusion in women undergoing hysterectomy for PAS.^(33,34)^

In terms of intraoperative safety, the management of PAS requires careful planning due to the complexity of the procedures and the high risk of visceral organ injuries. Urological complications occur in approximately 20% of hysterectomies performed for PAS,^(35)^ and massive blood loss remains both common and difficult to predict.^(36)^ In our analysis, injury and transfusion rates appeared non-inferior between groups; however, strategies to effectively mitigate these intraoperative challenges remain elusive.

Moreover, in the non-inferiority analysis, we found a high probability that conservative surgery is non-inferior to hysterectomy in terms of blood loss. However, we could not make the same claim for other critical outcomes, such as intraoperative visceral organ injury and long-term complications. This limitation is largely attributable to the low prevalence of these events, which resulted in underpowered analyses. Nonetheless, it is unlikely that a major increase in harm associated with conservative surgery would be detected.

## Conclusion

Conservative surgery in women diagnosed with placenta accreta spectrum disorders may not lead to higher blood loss, number of packs of red blood cells transfused, intraoperative visceral organ injury, and duration of surgery compared to hysterectomy. Nonetheless, this approach may not be advantageous in all women diagnosed with PAS, as specific placental topographies may lead to worse outcomes. Further research should seek to increase the total sample size of randomized trials comparing conservative surgery to hysterectomy in order to minimize imprecision on confidence interval boundaries and review information size.

## Data Availability

The research data are described in the article presented.

## References

[1] Matsuzaki S, Mandelbaum RS, Sangara RN, McCarthy LE, Vesta NL, Klar M (2021). Trends, characteristics, and outcomes of placenta accreta spectrum: a national study in the United States. Am J Obstet Gynecol.

[2] Sentilhes L, Kayem G, Chandraharan E, Palacios-Jaraquemada J, Jauniaux E (2018). FIGO Placenta Accreta Diagnosis and Management Expert Consensus Panel. FIGO consensus guidelines on placenta accreta spectrum disorders: Conservative management. Int J Gynaecol Obstet.

[3] Sentilhes L, Seco A, Azria E, Beucher G, Bonnet M-P, Branger B (2022). Conservative management or cesarean hysterectomy for placenta accreta spectrum: the PACCRETA prospective study. Am J Obstet Gynecol.

[4] Jaraquemada JMP, Pesaresi M, Nassif JC, Hermosid S (2004). Anterior placenta percreta: surgical approach, hemostasis and uterine repair. Acta Obstet Gynecol Scand.

[5] Page MJ, McKenzie JE, Bossuyt PM, Boutron I, Hoffmann TC, Mulrow CD (2021). The PRISMA 2020 statement: An updated guideline for reporting systematic reviews. PLOS Med.

[6] Sterne JAC, Savović J, Page MJ, Elbers RG, Blencowe NS, Boutron I (2019). RoB 2: a revised tool for assessing risk of bias in randomised trials. BMJ.

[7] Sterne JA, Hernán MA, Reeves BC, Savović J, Berkman ND, Viswanathan M (2016). ROBINS-I: a tool for assessing risk of bias in non-randomised studies of interventions. BMJ.

[8] Guyatt G, Zhao Y, Mayer M (2023). GRADE guidance 36: updates to GRADE's approach to addressing inconsistency. J Clin Epidemiol.

[9] Luo D, Wan X, Liu J, Tong T (2018). Optimally estimating the sample mean from the sample size, median, mid-range, and/or mid-quartile range. Stat Methods Med Res.

[10] Viechtbauer W (2010). Conducting meta-analyses in R with the metafor package. J Stat Softw.

[11] Balduzzi S, Rücker G, Schwarzer G (2019). How to perform a meta-analysis with R: a practical tutorial. Evid Based Ment Health.

[12] Schünemann H, Brożek J, Guyatt G, Oxman A (2013). Handbook for grading the quality of evidence and the strength of recommendations using the GRADE approach (updated October 2013).

[13] Zeng L, Brignardello-Petersen R, Hultcrantz M, Mustafa RA, Murad MH, Iorio A (2022). GRADE Guidance 34: update on rating imprecision using a minimally contextualized approach. J Clin Epidemiol.

[14] Jauniaux E, Ayres-de-Campos D, Langhoff-Roos J, Fox KA, Collins S (2019). FIGO Placenta Accreta Diagnosis and Management Expert Consensus Panel. FIGO classification for the clinical diagnosis of placenta accreta spectrum disorders. Int J Gynaecol Obstet Off Organ Int Fed Gynaecol Obstet.

[15] Nieto-Calvache ÁJ, Aryananda RA, Palacios-Jaraquemada JM, Cininta N, Grace A, Benavides-Calvache JP (2024). One-step conservative surgery vs hysterectomy for placenta accreta spectrum: a feasibility randomized controlled trial. Am J Obstet Gynecol MFM.

[16] Nieto-Calvache AJ, Palacios-Jaraquemada JM, Aryananda R, Basanta N, Aguilera R, Benavides JP (2023). How to perform the one-step conservative surgery for placenta accreta spectrum move by move. Am J Obstet Gynecol MFM.

[17] Paping A, Bluth A, Al Naimi A, Mhallem M, Kolak M, Jaworowski A (2025). Opportunities for, and barriers to, uterus-preserving surgical techniques for placenta accreta spectrum. Acta Obstet Gynecol Scand.

[18] Abouda HS, Aloui H, Azouz E, Marzouk SB, Frikha H, Hammami R (2025). New surgical technique for managing placenta accreta spectrum and pilot study of the "CMNT PAS" study. AJOG Glob Rep.

[19] van Beekhuizen HJ, Stefanovic V, Schwickert A, Henrich W, Fox KA, MHallem Gziri M (2021). A multicenter observational survey of management strategies in 442 pregnancies with suspected placenta accreta spectrum. Acta Obstet Gynecol Scand.

[20] Vuong AD, Pham TH, Pham XT, Truong DP, Nguyen XT, Trinh NB (2024). Modified one-step conservative uterine surgery (MOSCUS) versus cesarean hysterectomy in the management of placenta accreta spectrum: A single-center retrospective analysis based on 619 Vietnamese pregnant women. Int J Gynaecol Obstet.

[21] Aryananda RA, Duvekot JJ, Van Beekhuizen HJ, Cininta NI, Ariani G, Dachlan EG (2024). Transabdominal and transvaginal ultrasound findings help to guide the clinical management of placenta accreta spectrum cases. Acta Obstet Gynecol Scand.

[22] Aryananda RA, Aditiawarman A, Gumilar KE, Wardhana MP, Akbar MIA, Cininta N (2022). Uterine conservative-resective surgery for selected placenta accreta spectrum cases: Surgical-vascular control methods. Acta Obstet Gynecol Scand.

[23] Jafari RM, Najafian M, Barati M, Saadati N, Jalili Z, Poolad A (2022). Comparison of uterine preservation versus hysterectomy in women with placenta accreta: A cross-sectional study. Int J Reprod Biomed.

[24] Durukan H, Durukan ÖB, Yazıcı FG (2021). Placenta accreta spectrum disorder: a comparison between fertility-sparing techniques and hysterectomy. J Obstet Gynaecol J Inst Obstet Gynaecol.

[25] Üstünyurt E (2020). Local uterine resection with Bakri balloon placement in placenta accreta spectrum disorders. Turk J Obstet Gynecol.

[26] Kilicci C, Ozkaya E, Eser A, Bostanci EE, Sanverdi I, Abide Yayla C (2018). Planned cesarean hysterectomy versus modified form of segmental resection in patients with placenta percreta. J Matern Fetal Neonatal Med.

[27] Reeves B, Deeks J, Higgins J, Shea B, Tugwell P, Wells G Chapter 24: Including non-randomized studies on intervention effects. Cochrane Handbook for Systematic Reviews of Interventions version 6.4 (updated August 2023).

[28] Javinani A, Qaderi S, Hessami K, Shainker SA, Shamshirsaz AA, Fox KA (2024). Delivery outcomes in the subsequent pregnancy following the conservative management of placenta accreta spectrum disorder: a systematic review and meta-analysis. Am J Obstet Gynecol.

[29] Zhao H, Liu C, Fu H, Abeykoon SD, Zhao X (2023). Subsequent pregnancy outcomes and risk factors following conservative treatment for placenta accreta spectrum: a retrospective cohort study. Am J Obstet Gynecol MFM.

[30] Palacios-Jaraquemada JM, Basanta N, Labrousse C, Martínez M (2022). Pregnancy outcome in women with prior placenta accreta spectrum disorders treated with conservative-reconstructive surgery: analysis of 202 cases. J Matern-Fetal Neonatal Med Off J Eur Assoc Perinat Med Fed Asia Ocean Perinat Soc Int Soc Perinat Obstet.

[31] Nieto-Calvache AJ, Palacios-Jaraquemada JM, Hussein AM, Jauniaux E, Milani Coutinho C, Rijken M (2024). Management of placenta accreta spectrum in low- and middle-income countries. Best Pract Res Clin Obstet Gynaecol.

[32] Piñas-Carrillo A, Chandraharan E (2021). Conservative surgical approach: The Triple P procedure. Best Pract Res Clin Obstet Gynaecol.

[33] Bonsen LR, Sleijpen K, Hendriks J, Urlings TA, Dekkers OM, le Cessie S (2024). Prophylactic radiologic interventions for postpartum hemorrhage control in women with placenta accreta spectrum disorder. Obstet Gynecol.

[34] Yu SCH, Cheng YK, Tse WT, Sahota DS, Chung MY, Wong SS (2020). Perioperative prophylactic internal iliac artery balloon occlusion in the prevention of postpartum hemorrhage in placenta previa: a randomized controlled trial. Am J Obstet Gynecol.

[35] Lucidi A, Jauniaux E, Hussein AM, Coutinho CM, Tinari S, Khalil A (2023). Urological complications in women undergoing Cesarean section for placenta accreta spectrum disorders: systematic review and meta-analysis. Ultrasound Obstet Gynecol.

[36] Wright JD, Pri-Paz S, Herzog TJ, Shah M, Bonanno C, Lewin SN (2011). Predictors of massive blood loss in women with placenta accreta. Am J Obstet Gynecol.

