# Supplementary material for: Conservative surgery vs hysterectomy for placenta accreta spectrum disorders: a systematic review and meta-analysis
Source: Rev Bras Ginecol Obstet. 2026 Aug 20;48:e-rbgo45. doi: 10.61622/rbgo/2026rbgo45 (PMC13690400; doi:10.61622/rbgo/2026rbgo45)
Supplement: Supplementary file 1 [file 1806-9339-rbgo-48-e-rbgo45-Suppl01.pdf]

Supplementary Materials

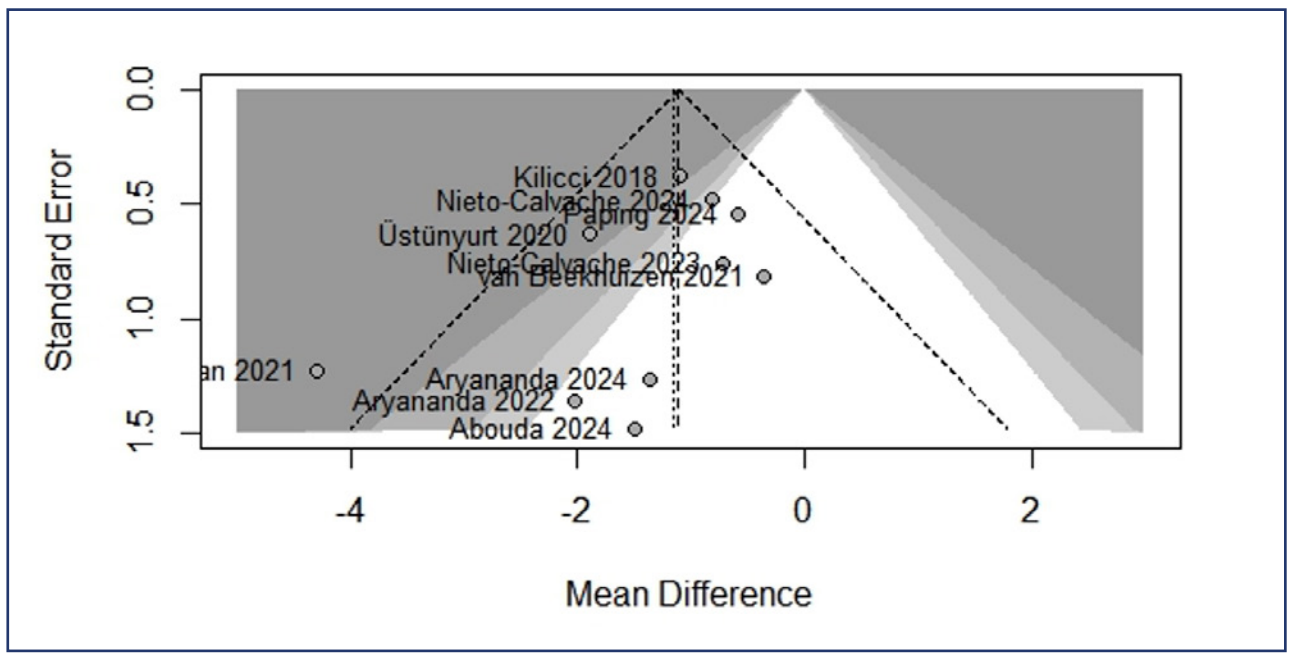

Figure S1. Funnel plot of the packs of red blood cells analysis

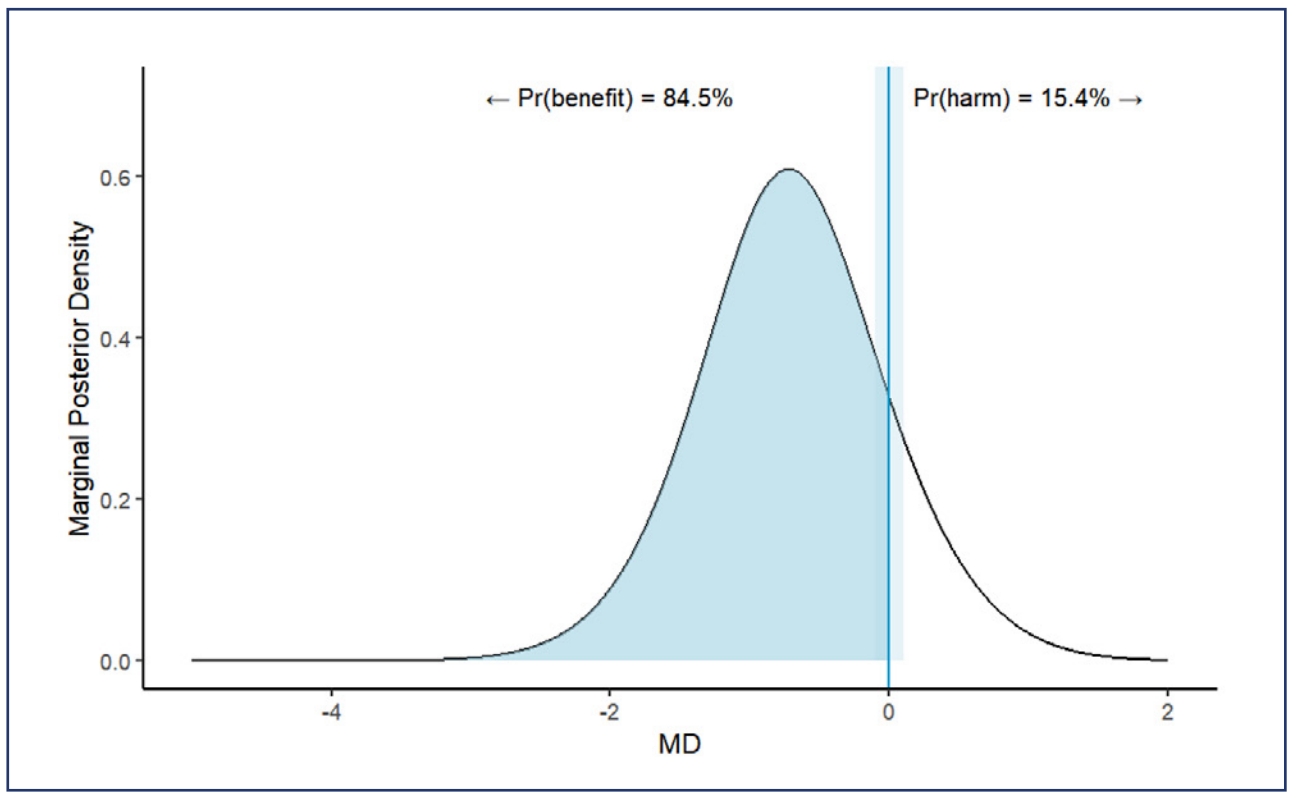

Figure S2. Posterior distribution of packs of red blood cells (pRBC) transfusions comparing conservative surgery to hysterectomy. There is an estimated probability that varies from 85.0% of reduced transfusion requirements to a 15.0% probability of increased requirements with conservative surgery. Assuming a non-inferiority margin of 1 pRBCs, the probability that conservative surgery is non-inferior to hysterectomy is 99.0%, while a threshold of 0.5 pRBCs has a probability of 95.4%. MD, mean difference

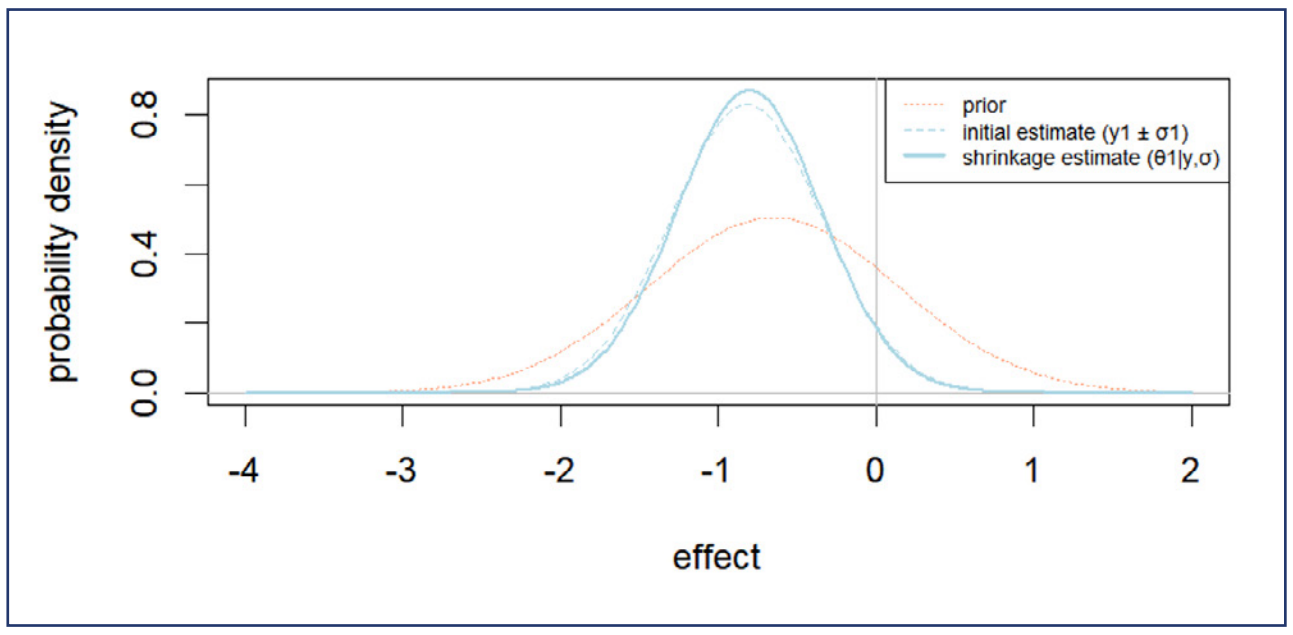

Figure S3. Prior, shrinkage and posterior distributions of the packs of red blood cells (pRBC) analysis, indicating minimal influence of the prior nonrandomized estimate on the final shrinkage estimate

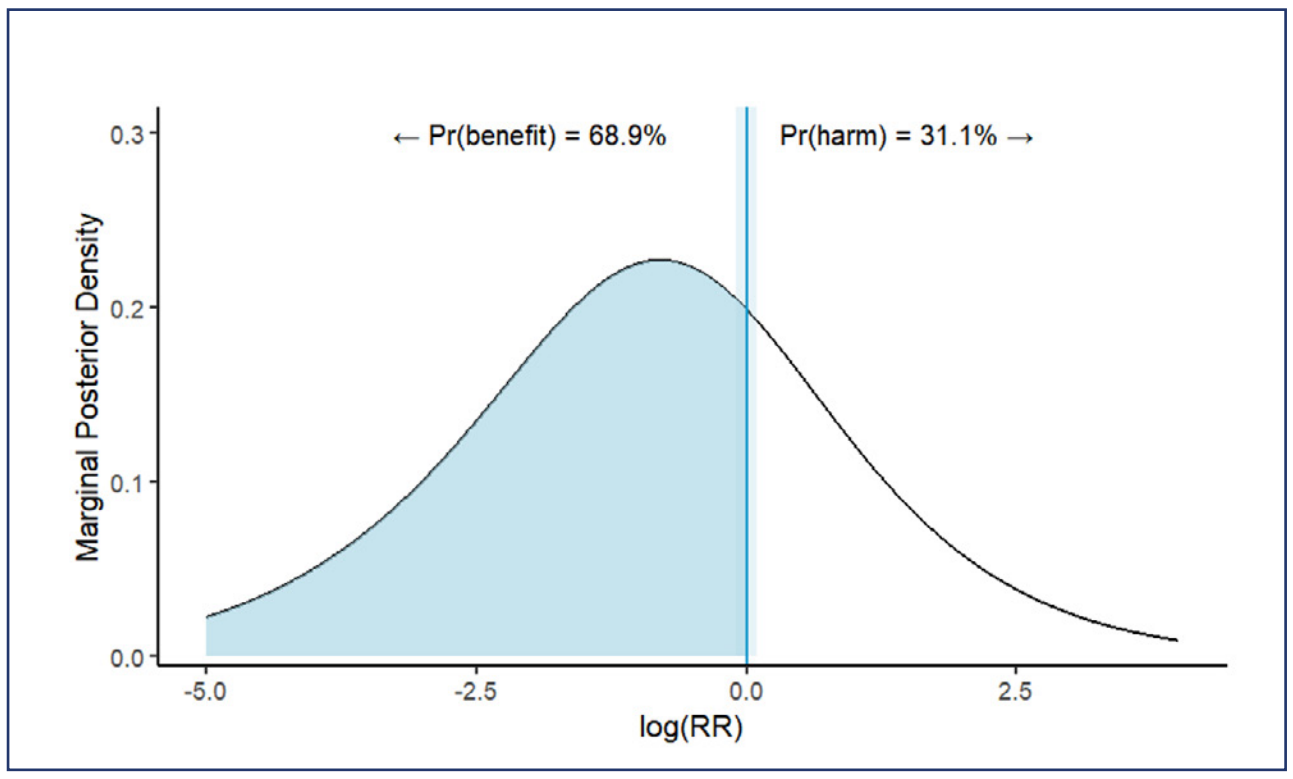

Figure S4. Posterior distribution of risk of bladder injury comparing conservative surgery to hysterectomy. There is an estimated probability that varies from 69% of reduced risk to a 31% probability of increased risk with conservative surgery. Assuming a non-inferiority margin of 1% absolute risk difference (RR 1.3), the probability that conservative surgery is non-inferior to hysterectomy is 71.5%. log(RR), risk ratio on the logarithm scale; Pr, probability

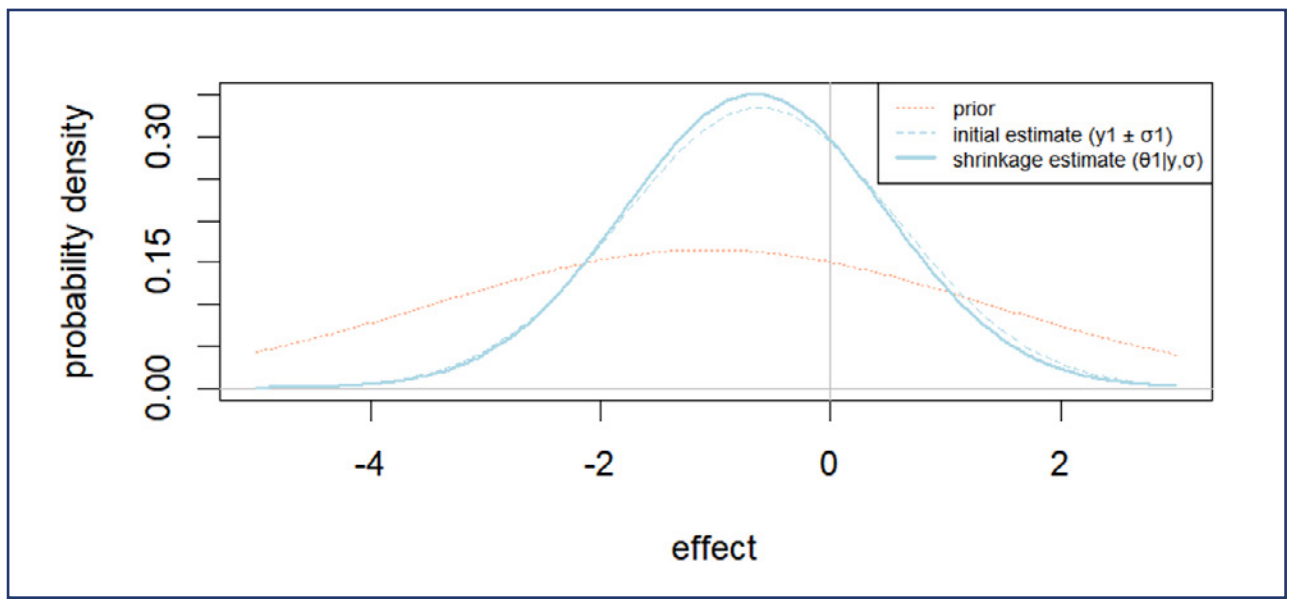

Figure S5. Prior, shrinkage and posterior distributions of the bladder injury analysis, indicating minimal influence of the prior nonrandomized estimate on the final shrinkage estimate

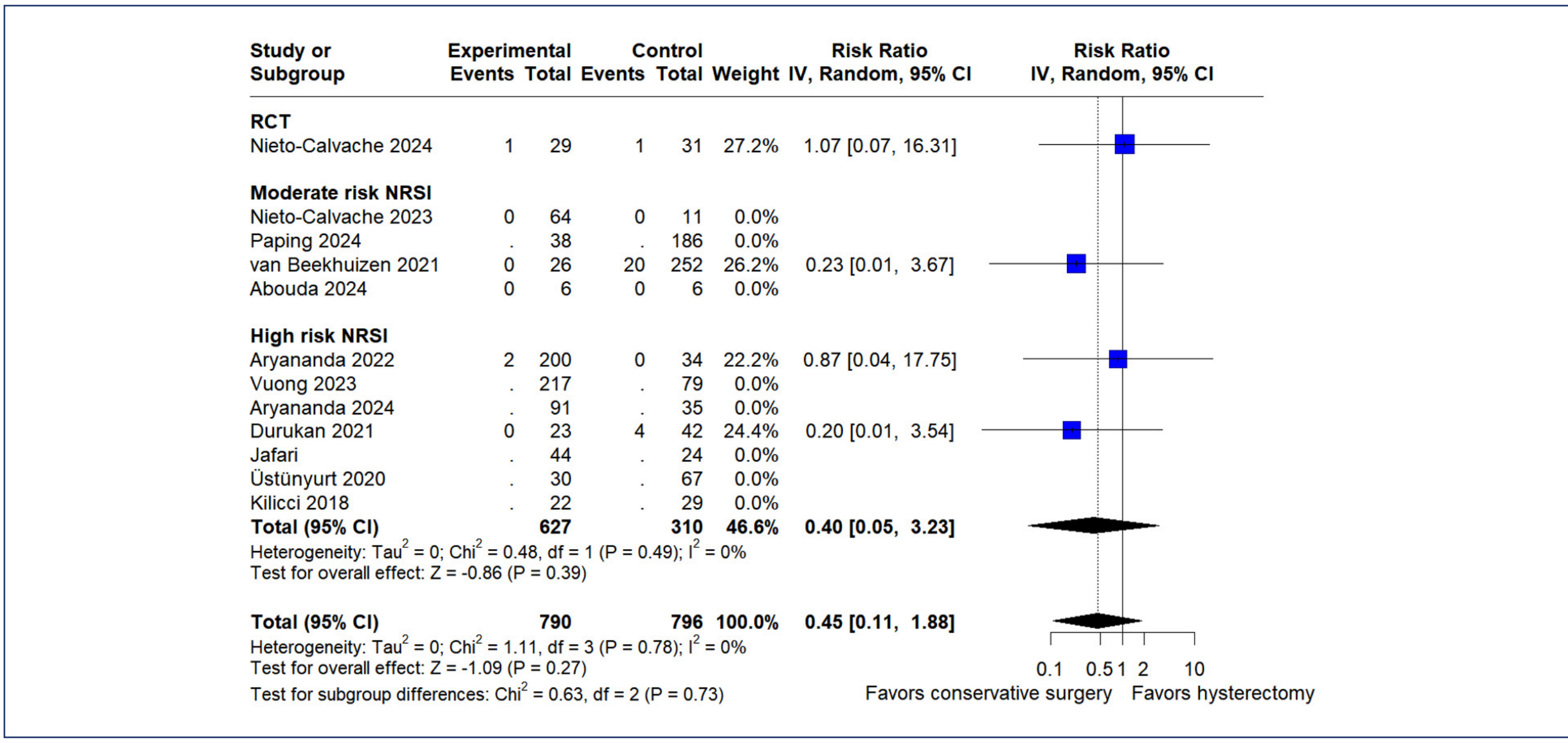

RCT - randomized controlled trial; IV - inverse variance; NRSI - nonrandomized study of intervention

Figure S6. Risk ratio of reoperation comparing conservative surgery to hysterectomy

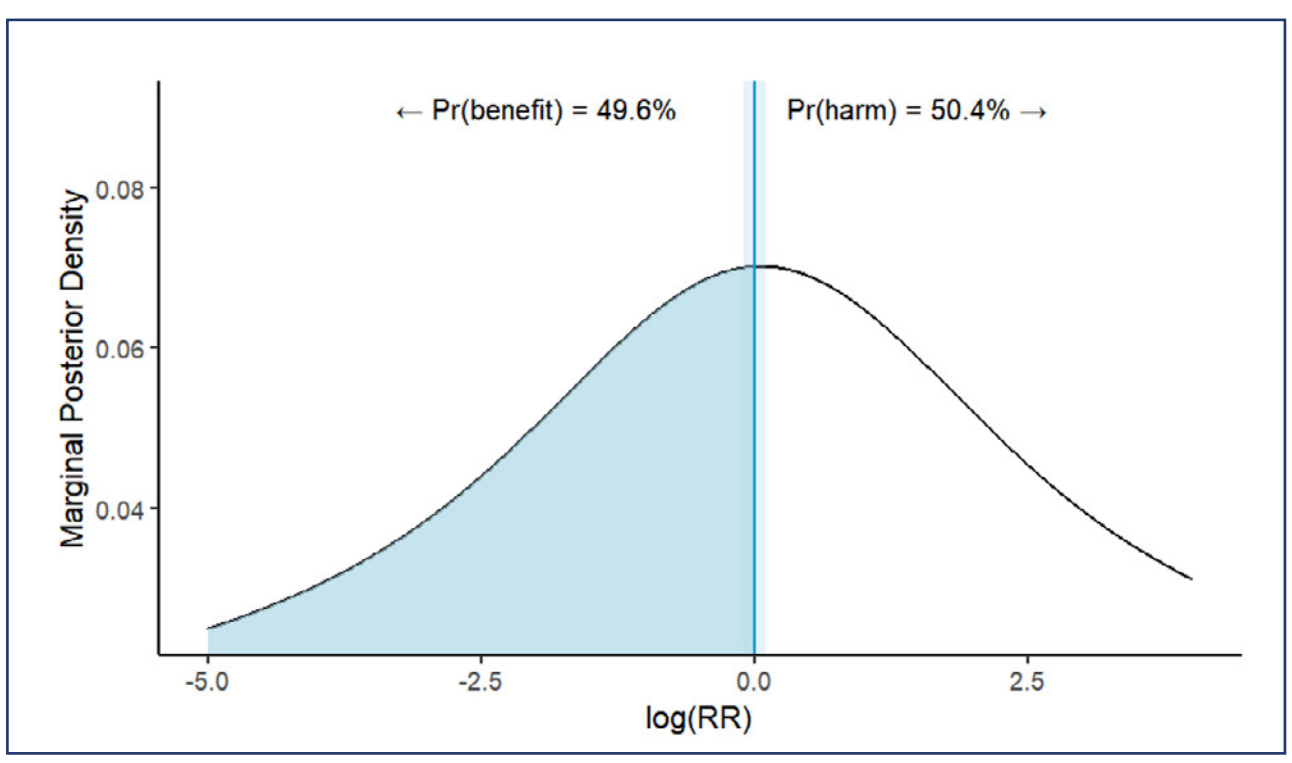

Figure S7. Posterior distribution of risk of reoperation comparing conservative surgery to hysterectomy. There is an estimated probability that varies from 49.6% of reduced risk to a 50.4% probability of increased risk with conservative surgery. Assuming a non-inferiority margin of 1% absolute risk difference (RR 1.3), the probability that conservative surgery is non-inferior to hysterectomy is 51.4%. log(RR), risk ratio on the logarithm scale

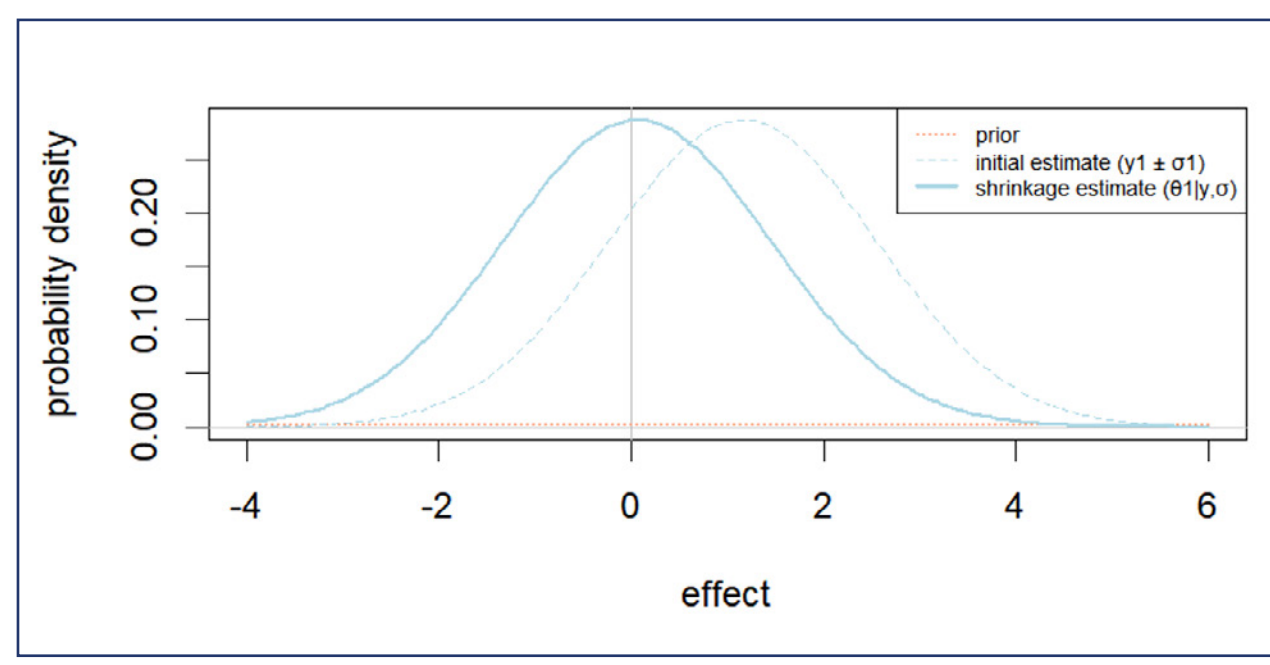

Figure S8. Prior, shrinkage and posterior distributions of the risk of reoperation analysis, indicating moderate influence of the prior nonrandomized estimate on the final shrinkage estimate

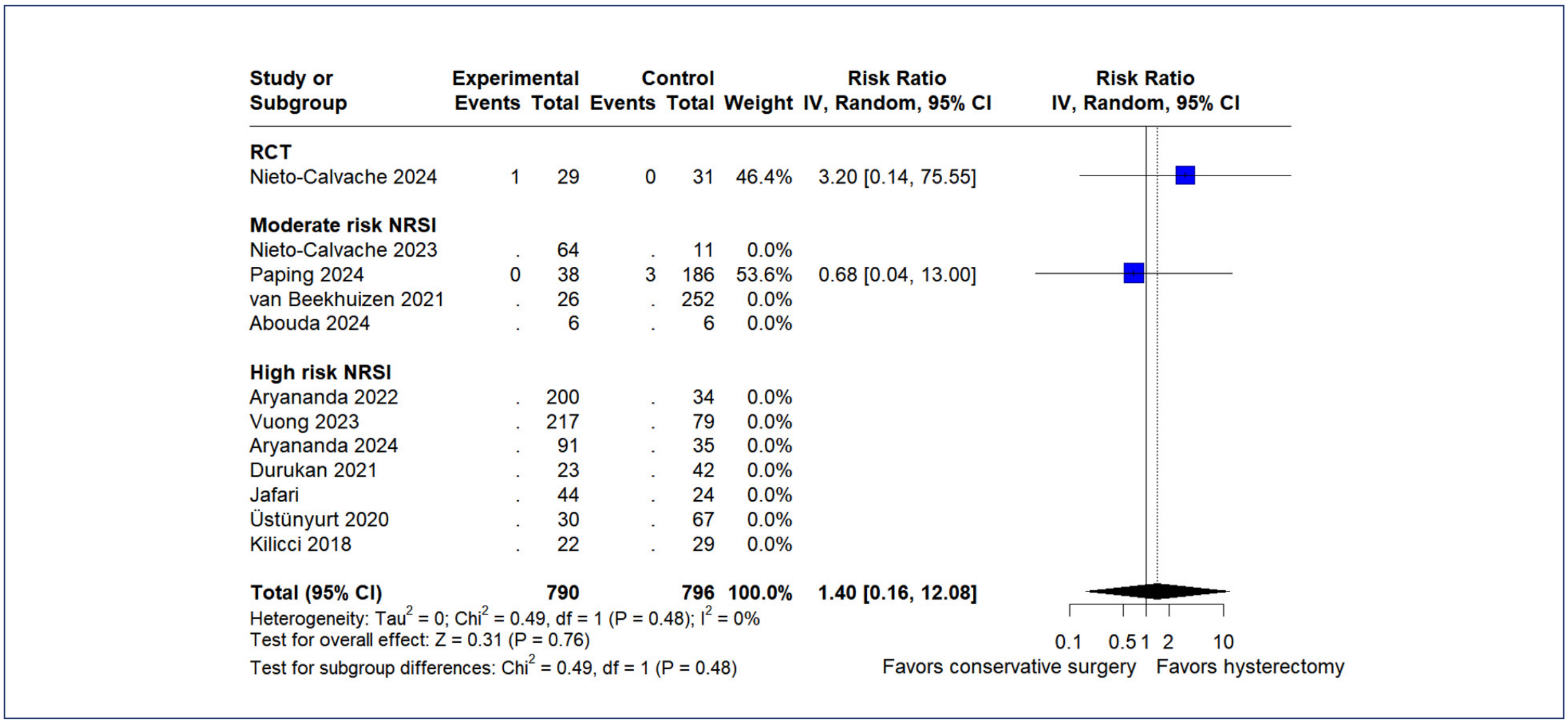

RCT - randomized controlled trial; IV - inverse variance; NRSI -nonrandomized study of intervention

Figure S9. Risk ratio of fistula formation comparing conservative surgery to hysterectomy

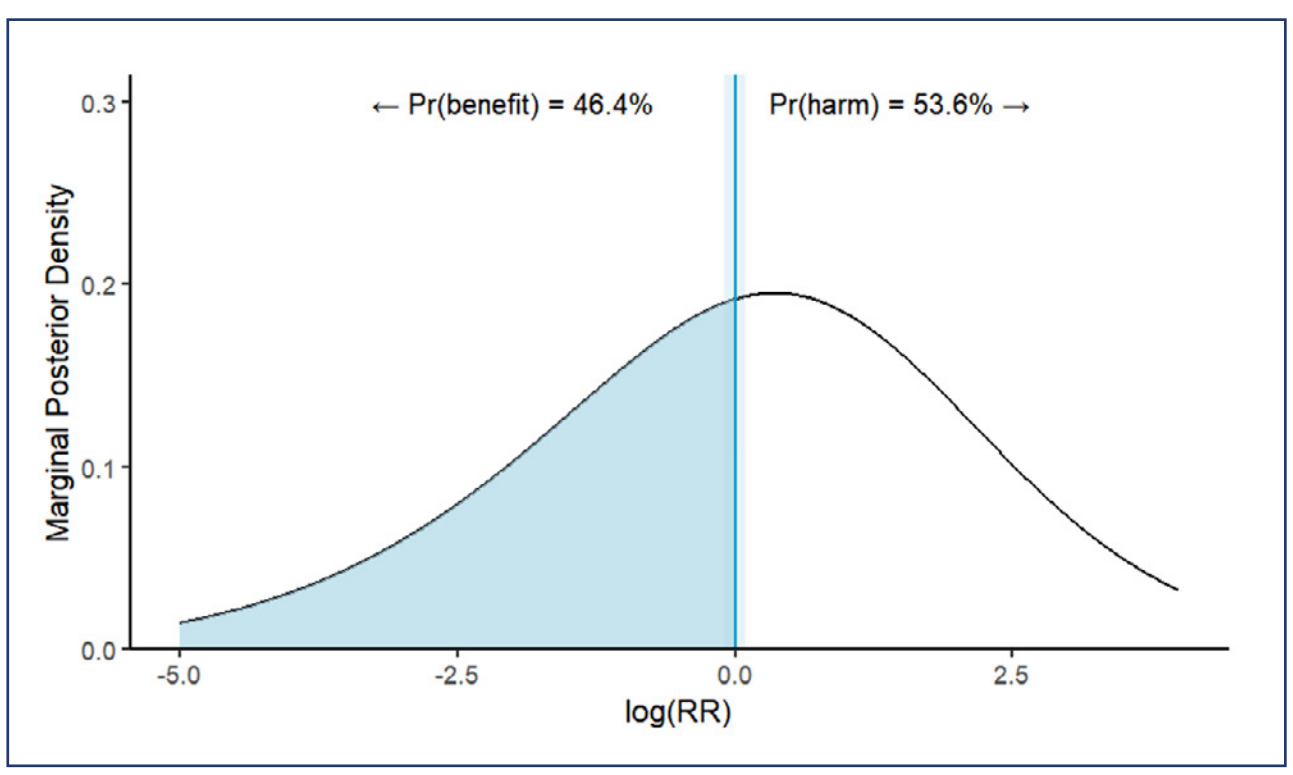

Figure S10. Posterior distribution of fistula formation comparing conservative surgery to hysterectomy. There is an estimated probability that varies from 46.4% of reduced risk to a 53.6% probability of increased risk with conservative surgery. Assuming a non-inferiority margin of 15% increased risk (risk difference not estimable), the probability that conservative surgery is non-inferior to hysterectomy is 49.0%. log(RR), risk ratio on the logarithm scale

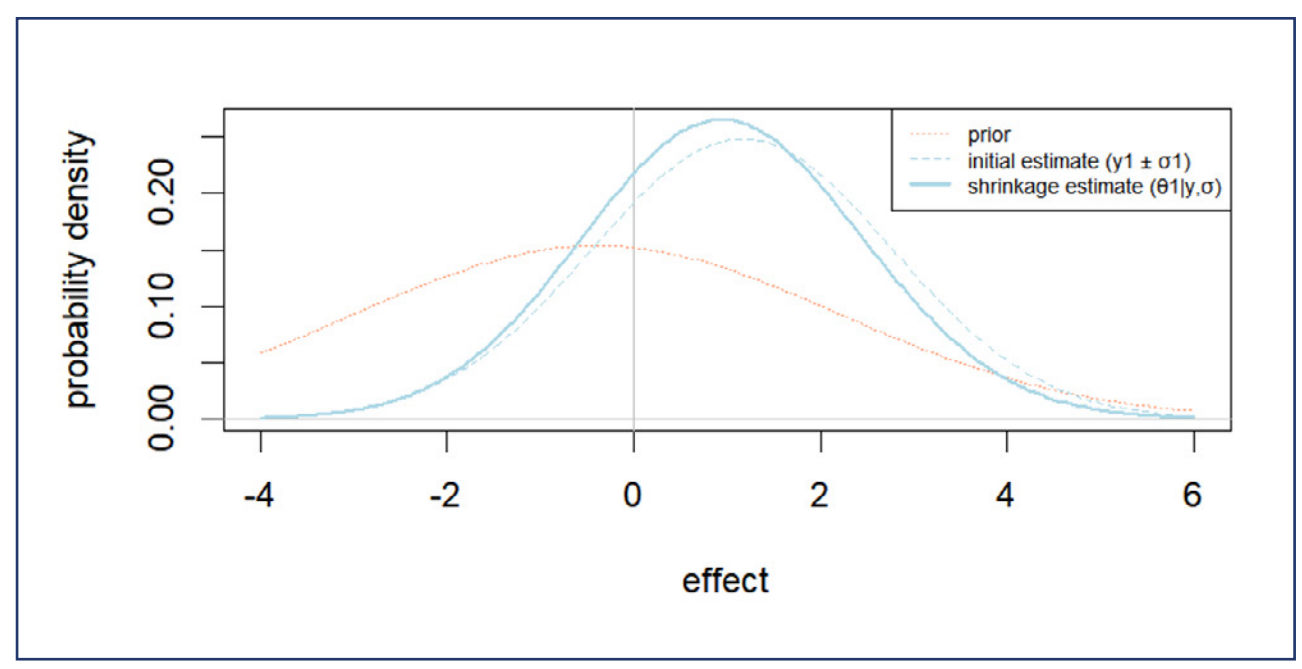

Figure S11. Prior, shrinkage and posterior distributions of the fistula formation analysis, indicating some influence of the prior nonrandomized estimate on the final shrinkage estimate

Supplementary Material 1. Search strategy

**Medline through PubMed**
Query: (one step conservative surgery OR uterine conservative-resective surgery OR uterus resection OR uterine resective surgery OR uterine conservative surgery OR uterus conservation surgery OR uterine preserving surgery OR fertility sparing surgery OR fertility preserving surgery OR triple p procedure OR triple p) and (placenta accreta spectrum OR placenta increta OR placenta percreta OR abnormally invasive placenta OR morbidly adherent placenta)
**Embase**
Query: ('placenta accreta spectrum'/exp OR 'morbidly adherent placenta' OR 'morbidly adherent placentas' OR 'placenta accreta spectrum' OR 'placenta accreta spectrum disorder' OR 'placenta accreta spectrum disorders' OR 'placenta acreta spectrum') AND ('one step surgery' OR 'triple p procedure' OR 'uterine resective surgery' OR 'uterine resective surgery' OR 'uterine conservative surgery' OR 'uterine preserving surgery' OR 'fertility sparing surgery'/exp OR 'fertility preserving surgery')
**Cochrane Library**
Query: (one step conservative surgery OR uterine conservative-resective surgery OR uterus resection OR uterine resective surgery OR uterine conservative surgery OR uterus conservation surgery OR uterine preserving surgery OR fertility sparing surgery OR fertility preserving surgery OR triple p procedure OR triple p) and (placenta accreta spectrum OR placenta increta OR placenta percreta OR abnormally invasive placenta OR morbidly adherent placenta)
